# The 2‐Amino Group of 8‐Aza‐7‐deaza‐7‐bromopurine‐2,6‐diamine and Purine‐2,6‐diamine as Stabilizer for the Adenine–Thymine Base Pair in Heterochiral DNA with Strands in Anomeric Configuration

**DOI:** 10.1002/chem.202004221

**Published:** 2020-12-21

**Authors:** Yingying Chai, Dasharath Kondhare, Aigui Zhang, Peter Leonard, Frank Seela

**Affiliations:** ^1^ Laboratory of Bioorganic Chemistry and Chemical Biology Center for Nanotechnology Heisenbergstrasse 11 48149 Münster Germany; ^2^ Laboratorium für Organische und Bioorganische Chemie Institut für Chemie neuer Materialien Universität Osnabrück Barbarastrasse 7 49069 Osnabrück Germany; ^3^ Department of Respiratory Critical Care Medicine Targeted Tracer, Research and Development Laboratory West China Hospital Sichuan 610041 P. R. China

**Keywords:** configuration, heterochiral, parallel DNA, purine-2,6-diamine, pyrazolo[3,4-*d*]pyrimidine

## Abstract

Stabilization of DNA is beneficial for many applications in the fields of DNA therapeutics, diagnostics, and materials science. Now, this phenomenon is studied on heterochiral DNA, an autonomous DNA recognition system with complementary strands in α‐D and β‐D configuration showing parallel strand orientation. The 12‐mer heterochiral duplexes were constructed from anomeric (α/β‐D) oligonucleotide single‐strands. Purine‐2,6‐diamine and 8‐aza‐7‐deaza‐7‐bromopurine‐2,6‐diamine 2′‐deoxyribonucleosides having the capability to form tridentate base pairs with dT were used to strengthen the stability of the dA–dT base pair. *T*_m_ data and thermodynamic values obtained from UV melting profiles indicated that the 8‐aza‐7‐deaza 2′‐deoxyribonucleoside decorated with a bromo substituent is so far the most efficient stabilizer for heterochiral DNA. Compared with that, the stabilizing effect of the purine‐2,6‐diamine 2′‐deoxyribonucleoside is low. Global changes of helix structures were identified by circular dichroism (CD) spectra during melting.

## Graphical abstract

**Base pair stabilization**: Heterochiral parallel duplexes represent an autonomous recognition system constructed from anomeric (α/β‐D) oligonucleotides. The naturally occurring purine‐2,6‐diamine and the artificial 8‐aza‐7‐deaza‐7‐bromopurine nucleosides **1** and **2** strengthen the dA–dT base pair. The 8‐aza‐7‐deazapurine nucleoside decorated with a bromo substituent is so far the most efficient stabilizer for this pairing system.

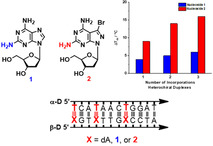

## Introduction

Modified nucleosides, if naturally occurring, bioinspired, or totally artificial, represent an important class of molecules with valuable properties as monomeric compounds or as constituents of DNA and RNA. They are beneficial to generate new nucleic acid structures, to identify binding sites, and to broaden the applicability of nucleic acids in the field of therapeutics, diagnostics, and material science.^[1]^

**Figure 1:**
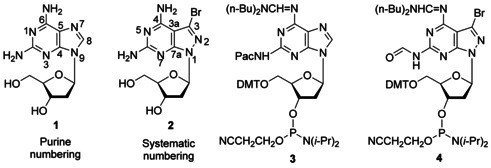
Structures of 2,6‐diaminonucleosides and their phosphoramidite building blocks. For full names of compounds, see the Experimental Section, General methods and materials.

**Figure 2:**
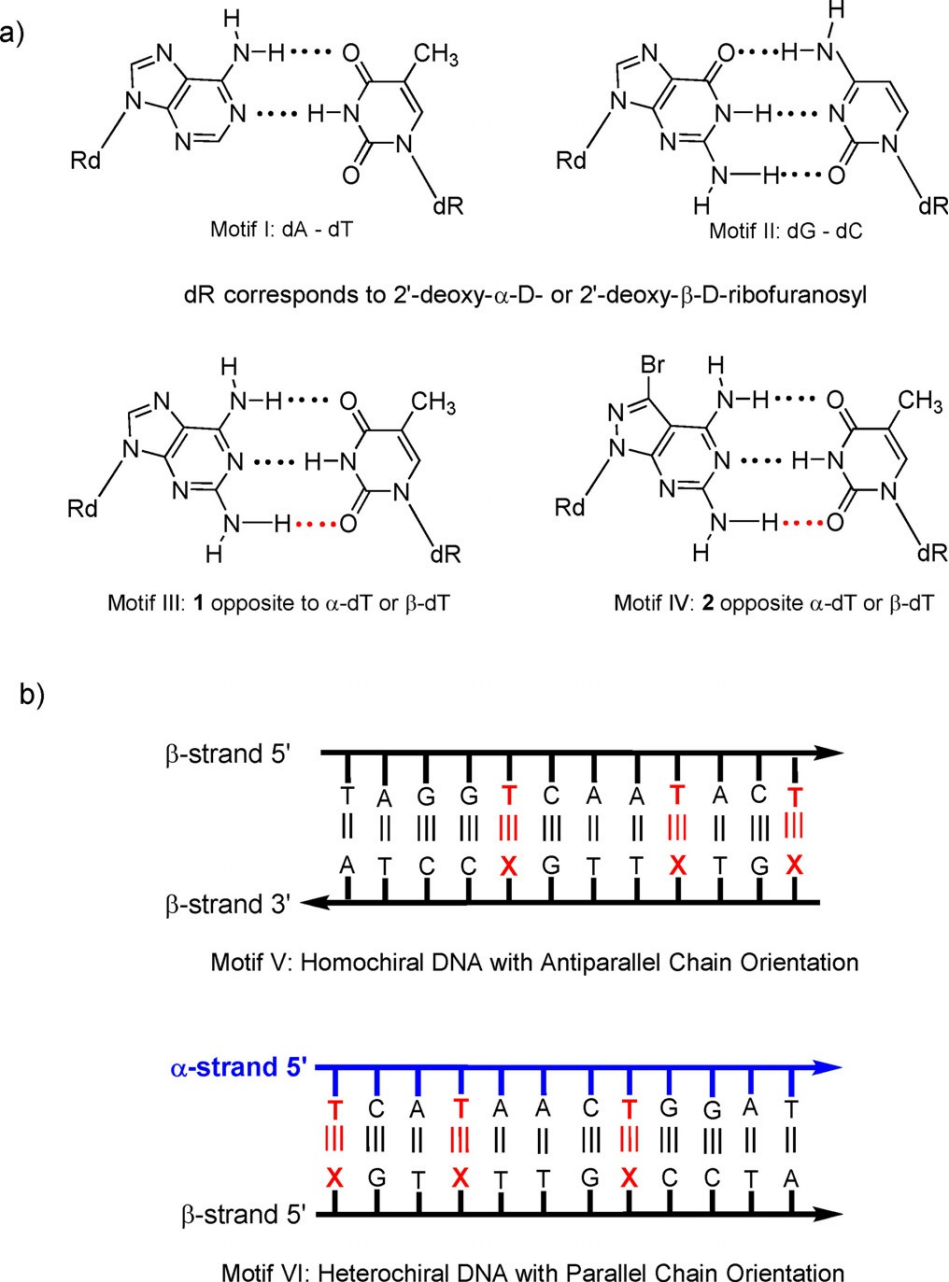
a) Base pairing motifs of **1** and **2** as well as dA–dT and dG–dC. Red dots indicate possible hydrogen bonds. b) Schematic representation of the alignments of oligonucleotide strands of homochiral and heterochiral duplexes. **X** represents dA, **1**, or **2**.

The purine‐2,6‐diamine nucleoside **1**[^2a^, ^2b^] is a naturally occurring DNA constituent replacing dA in the Cyanophage S‐2L (Figure 1).^[2c]^ It offers a way to change the chemical, physical, and biological properties of DNA, when replacing dA in the dA–dT base pair (purine numbering is used throughout the manuscript; Figure 2 a).^[3]^ Compound **1** is anticipated to form a third hydrogen bond with dT in a similar way as observed for the dG–dC pair.^[4]^ However, the situation is more complex than expected, and experiments indicate that the stabilizing effect by the additional 2‐amino group is less efficient than in the dG–dC pair. This differs in double helical DNA and RNA.^[4]^ In DNA, the purine‐2,6‐diamine‐thymine base pair can be more stable, equally stable, or less stable than the adenine–thymine pair. This depends on the sequence of the duplex. The phenomenon has been reported;^[5]^ however, it found not much attention in the realm of nucleic acid chemistry and biology.

Our laboratory has developed a purine‐2,6‐diamine analog with a pyrazolo[3,4‐*d*]pyrimidine (8‐aza‐7‐deazapurine) skeleton decorated with a bromo atom in position‐7 (**2**; Figure 1).^[6]^ Nucleoside **2** increases the dA–dT base pair stability substantially, when replacing dA in homochiral DNA (Figure 2 a, motif IV).^[6]^ As a consequence, the “dA–dT” pair becomes as stable as the dG–dC pair.^[6d]^ This modification makes the base pair stability independent of the content of dA–dT and dG–dC pairs, leading to a harmonization of DNA stability, which is essential for the correct hybridization of oligonucleotides on biochips and in solution.^[7]^

Recently, we reported on the structural perturbation of heterochiral DNA (Figure 2 b). Here, heterochiral DNA means that two diastereoisomeric strands with α‐D and β‐D configuration and not enantiomeric oligonucleotides as in d‐/l‐DNA form the double helix (Figure 2 b, motif VI).^[8]^ The heterochiral DNA forms parallel chains,^[9]^ displays a global helical B‐type structure similar to canonical DNA, and represents an autonomous recognition system beneficial to various fields in nucleic acid chemistry, biology, and for the construction of nanodevices. Early attempts to stabilize heterochiral DNA used the α‐anomer of 5‐propynyl‐2′‐deoxycytidine,^[10]^ the α‐D anomer of **1**,^[11]^ and a LNA derivative.^[12]^ However, the stability increase was low in all cases.

From this background, we wanted to study the base pairing of 8‐aza‐7‐deaza‐7‐bromopurine‐2,6‐diamine nucleoside **2** with dT in heterochiral DNA. More specific, the stabilization of the 2‐amino group of nucleoside **2** with dT was investigated on 12‐mer duplexes. For comparison, base pair stability of the purine‐2,6‐diamine nucleoside **1** was determined. Modifications executed on heterochiral DNA were also performed on homochiral DNA and stabilization was compared. To this end, phosphoramidite building blocks **3** and **4** were synthesized (Figure 1). As for the synthesis of **3**, a patent application only describes a laborious multi‐step procedure,^[13]^ we herein report a short and efficient synthesis route with full characterization of all compounds. Oligonucleotides containing increasing numbers of **1** and **2** were prepared. Thermal duplex stability was determined by *T*_m_ measurements, thermodynamic data of base pairs were calculated, and circular dichroism (CD) spectra were measured to detect global helical changes.

## Results and Discussion

### Syntheses and properties of monomers

So far, tridentate base pair formation with the 2,6‐diamino nucleosides **1** or **2** has been studied in homochiral DNA, DNA/RNA hybrids, RNA as well as other nucleic acid analogs with a conventional antiparallel alignment of the strands.[^4^, ^5^, ^14^] In this work, both compounds were transferred to DNA with a parallel strand arrangement.

The 8‐aza‐7‐deaza‐7‐bromopurine‐2,6‐diamine nucleoside **2** and purine analog **1** display special chemical and physical properties with respect to the dA. Compound **2** is significantly more stable at the glycosylic bond than the very labile **1**.^[6a]^ Also, the p*K*_a_ values and reactivity for the 2‐ and 6‐amino groups of **1** and **2** differ significantly. The p*K*_a_ value of **1** was determined to be 4.6, whereas the pyrazolo[3,4‐*d*]pyrimidine nucleoside **2** shows a p*K*_a_ of 3.7 similar to that of dA (3.6)^[15]^ (Figure S16 in the Supporting Information). The different p*K*_a_ values affect the protecting group stability. Furthermore, the UV spectra of **1** and **2** display UV maxima at approximately 260 and 280 nm with only marginal shifts (4–5 nm), whereas dA shows only one absorption maximum at 260 nm (Figure S17 in the Supporting Information). These UV changes have an impact on the CD spectra, which will be discussed later.

Protecting groups for the building block synthesis of the diamino nucleosides **1** and **2** have to be carefully chosen, as their 2‐ and 6‐amino groups possess different reactivity. For compound **2**, a combination of a formyl group for 2‐amino protection and a dibutylamidine residue for the 6‐amino group was found to be the optimal combination.^[6a]^ This provides the 6‐amino protecting group sufficient stability, and the 2‐amino formyl protection is labile enough to be removed. Both protecting groups are released in concentrated aqueous NH_3_ at 55 °C within 12 h.

**Scheme 1:**
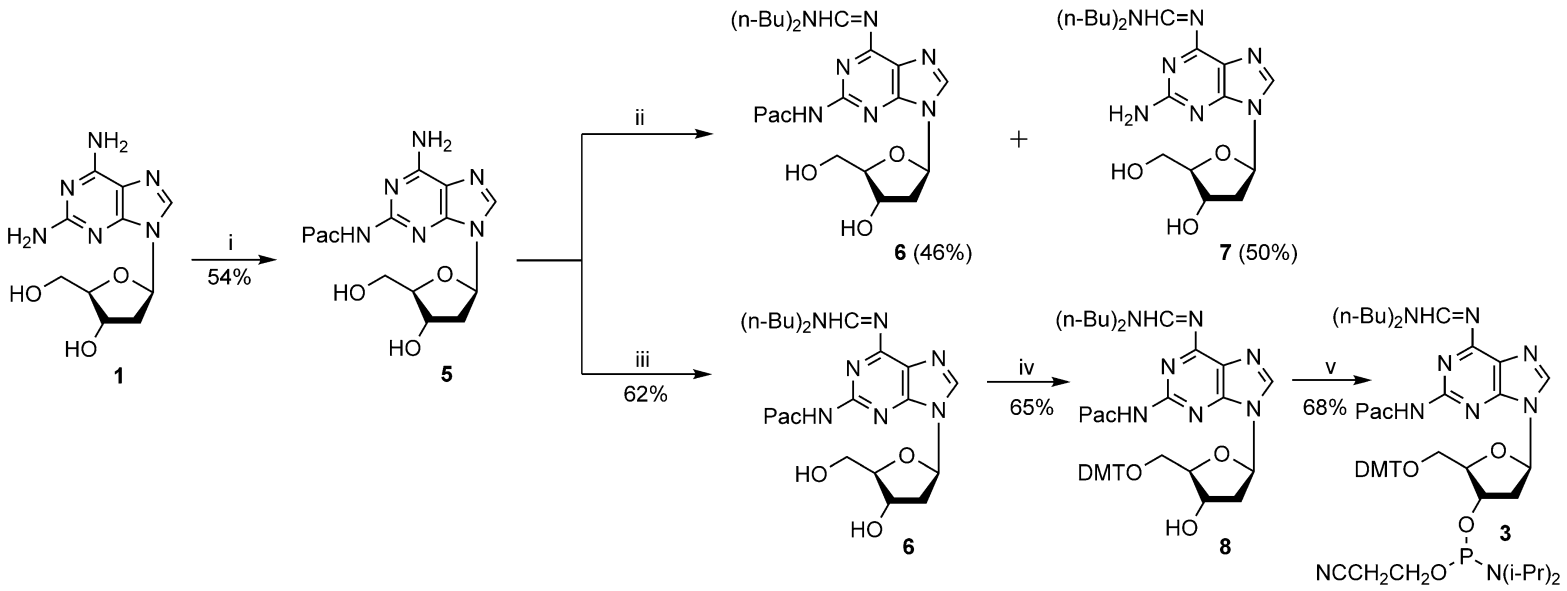
Synthesis of phosphoramidite **3**. Reagents and conditions: (i) a) trimethylsilyl chloride, pyridine, phenoxyacetyl chloride, 4 °C; b) MeOH/THF/28 % aq. NH_3_ (1:1:1), 1 h, rt; (ii) *N*,*N*‐dibutylformamide dimethylacetal, MeOH, 40 °C, 2 h; (iii) *N*,*N*‐dibutylformamide dimethylacetal, DMF, rt; (iv) 4,4′‐dimethoxytrityl chloride, pyridine, rt; (v) NC(CH_2_)_2_OP(Cl)N(*i*Pr)_2_, DIPEA (*N*,*N*‐diisopropylethylamine), CH_2_Cl_2_, rt.

For nucleoside **1** containing a labile *N*‐glycosylic bond, which is further destabilized by electron‐withdrawing substituents or electron‐withdrawing protecting groups, a substantial amount of literature has been accumulated to find the best combination for protection.[^4c^, ^4d^, ^4f^, ^13^, ^14d^, ^16^, ^17^] Isobutyryl groups^[14d]^ were found to be problematic as the 2‐amino protecting group was difficult to remove from oligonucleotides. An improved protecting group strategy^[13]^ for **1** employs an analogous protecting group combination (Pac, amidine) as used for nucleoside **2** (formyl, amidine).^[6a]^ However, as additional protection and deprotection steps are necessary in the literature protocol,^[13]^ a more efficient and shorter route with full characterization of intermediates is presented (Scheme 1). First, transient protection conditions^[18]^ and phenoxyacetyl chloride as acylating reagent were used to give the mono phenoxyacetyl derivative **5** in 54 % yield. The use of trichlorophenyl phenoxyacetate for the same purpose was reported but no experimental procedure was given.^[4d]^ A bis‐phenoxyacetylated derivative as reported earlier[^4c^, ^16a^] was not obtained, possibly owing to the instability of the 6‐amino protecting group. Then, compound **5** was treated with dibutylformamide dimethylacetal in DMF to yield the dibutylaminomethylidene derivative **6** in 62 %. When the reaction was performed in methanol, the 2‐phenoxyacetyl groups were partially lost and a mixture of **6** (46 %) and **7** (50 %) was isolated. Next, the 5′‐hydroxyl group of **6** was blocked with a DMT (4,4’‐dimethoxytrityl)‐residue (→**8**, 65 %) and the 3′‐OH phosphitylated under standard conditions affording **3** (68 %). Both amino protecting groups can be removed by conc. aq. NH_3_ and in addition the N6‐dibutylamidine group stabilizes the *N*‐glycosylic bond.[^16b^, ^17^, ^19^]

The synthesized compounds were characterized by ^1^H, ^13^C NMR spectra and ESI‐TOF mass spectra (see the Experimental Section). ^1^H‐^13^C correlated (HMBC and HSQC) NMR spectra were used to assign the ^13^C NMR signals. For details, see the Supporting Information (Table S1, Figures S21–46).

### Synthesis and base pair stabilization of heterochiral and homochiral DNA by nucleosides 1 and 2

To study the impact of the 2‐amino groups of nucleosides **1** and **2** as constituents of heterochiral and homochiral DNA, 12‐mer oligonucleotides (ODNs) were synthesized.

Standard oligonucleotide synthesis conditions were employed and conventional phosphoramidite building blocks as well as the phosphoramidites **3** and **4** were used. For homochiral DNA, complementary β‐strands were constructed and the duplex β‐5′‐d(TAGGTCAATACT) (ODN‐**1**)**⋅**β‐3′‐d(ATCCAGTTATGA) (ODN‐**2**) was used as a reference compound showing antiparallel chain orientation. For the construction of the parallel stranded heterochiral duplexes, in one of the two strands, all β‐d‐nucleosides, were replaced by their α‐D anomers resulting in the heterochiral duplex α‐5′‐d(TCATAACTGGAT) (ODN‐**3**)**⋅**β‐5’‐d(AGTATTGACCTA) (ODN‐**2**). The coupling yields of the modified building blocks were higher than 95 %. After solid‐phase synthesis, the oligodeoxyribonucleotides were cleaved from the solid support. Oligonucleotides were deprotected in 28 % aq. NH_3_ (55 °C, overnight), purified by reversed‐phase HPLC (RP‐18), detritylated with 2.5 % dichloroacetic acid in dichloromethane, and further purified by HPLC. Using phosphoramidite **3** or **4**, no depurination was observed during synthesis and deprotection of oligonucleotides. HPLC purity profiles of oligonucleotides are shown in the Supporting Information (Figures S1–S11). All oligonucleotides used in this study, and their masses determined by MALDI‐TOF mass‐spectrometry are listed in Table 1.

**Table 1:** Synthesized oligonucleotides and their molecular masses determined by MALDI‐TOF mass spectrometry.

Entry	Oligonucleotide	*M*_r_ calcd^[a]^ *M*_r_ found^[b]^
ODN**‐1**	β‐5′‐d(TAGGTC**A**ATACT)^[20]^	–
		
ODN**‐2**	β‐5′‐d(AGTAT**T**GACCTA)^[20]^	–
		
ODN**‐3**	α‐5′‐d(**T**CA**T**AAC**T**GGAT)^[8]^	3644.4 3644.0
ODN**‐4**	β‐5′‐d(AGT**1**TTGACCTA)	3659.4 3660.3
ODN**‐5**	β‐5′‐d(AGTATTG**1**CCTA)	3659.4 3658.4
ODN**‐6**	β‐5′‐d(AGT**1**TTG**1**CCTA)	3674.4 3675.7
ODN**‐7**	β‐5′‐d(**1**GT**1**TTG**1**CCTA)	3689.4 3689.2
ODN**‐8**	β‐5′‐d(AGT**2**TTGACCTA)	3738.3 3738.3
ODN**‐9**	β‐5′‐d(AGTATTG**2**CCTA)	3738.3 3738.1
ODN**‐10**	β‐5′‐d(AGT**2**TTG**2**CCTA)	3832.2 3832.4
ODN**‐11**	β‐5′‐d(**2**GT**2**TTG**2**CCTA)	3926.1 3927.7
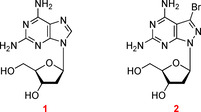		

[a] Calculated on the basis of the molecular mass of [*M*+H]^+^. [b] Determined by MALDI‐TOF mass‐spectrometry as [*M*+H]^+^ in the linear positive mode. **1** corresponds to purine‐2,6‐diamine 2′‐deoxyribonucleoside. **2** corresponds to 8‐aza‐7‐deaza‐7‐bromopurine‐2,6‐diamine 2′‐deoxyribonucleoside.^[6a]^

**Figure 3:**
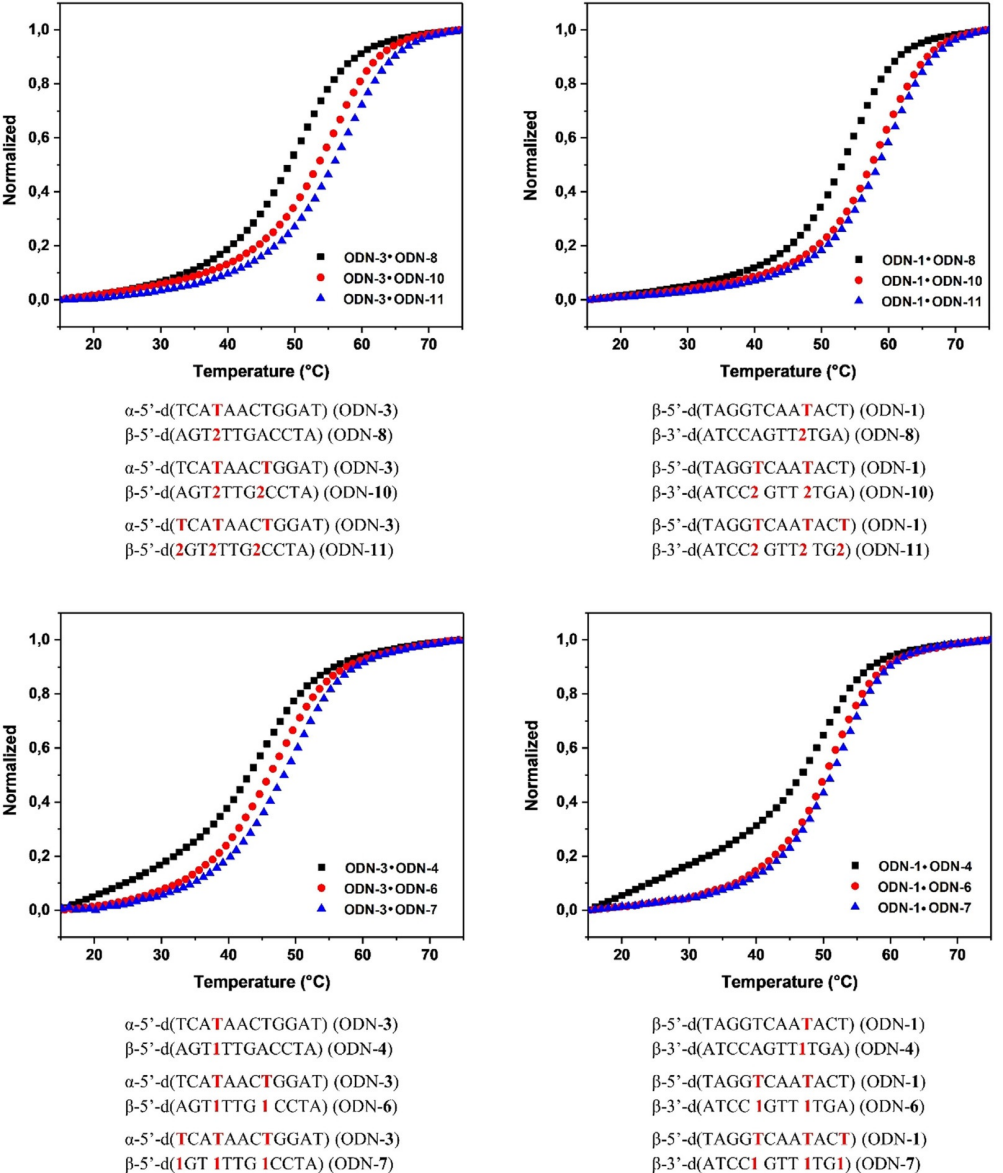
Thermal melting curves of heterochiral and homochiral duplexes monitored at 260 nm in 100 mm NaCl, 10 mm MgCl_2_, 10 mm Na‐cacodylate, pH 7.0 with 5 μm + 5 μm single‐strand concentration.

Oligonucleotides with the 8‐aza‐7‐deaza‐7‐bromopurine‐2,6‐diamine nucleoside **2** or the purine‐2,6‐diamine nucleoside **1** were hybridized with complementary strands displaying nucleoside residues either in α‐D or β‐D configuration. Melting curves of heterochiral and homochiral duplexes determined in 0.1 m NaCl, 10 mm MgCl_2_, 10 mm Na‐cacodylate buffer at 260 nm are shown in Figure 3. All melting curves showed strong cooperativity and duplexes were much more strongly stabilized by nucleoside **2** than **1** (Figure 3).

The number of dA replacements was identical for **1** and **2** and was increasing from one to three. As nucleosides **1** and **2** were also incorporated at identical positions, *T*_m_ data are comparable. In fact, both nucleosides stabilize homochiral and heterochiral DNA, but the effect is very low in the case of **1** compared with the strong stabilization induced by **2** (Table 2). The small *T*_m_ increase observed for the third incorporation of either **1** or **2** near the terminus of the double helix is related to fraying ends. This phenomenon, well‐known for homochiral DNA, is now documented for heterochiral DNA.

**Table 2:** *T*_m_ values and thermodynamic data of heterochiral or homochiral oligonucleotide duplexes containing purine‐2,6‐diamine nucleoside **1** or 8‐aza‐7‐deaza‐7‐bromopurine‐2,6‐diamine nucleoside **2**.^[a]^

	*T*_m_ [°C]	Δ*T*_m_ [°C]	Δ*H*_310_ [kcal mol^−1^]	Δ*S*_310_ [cal K^−1^ mol^−1^]	Δ*G*_310_ [kcal mol^−1^]
**Heterochiral Duplexes Parallel Strands**					
α‐5′‐d(TCATAACTGGAT) (ODN‐**3**) β‐5′‐d(AGTATTGACCTA) (ODN‐**2**)	41		−65	−180	−9.1
α‐5′‐d(TCATAACTGGAT) (ODN‐**3**) β‐5′‐d(AGT1TTGACCTA) (ODN‐**4**)	45	+4	−76	−213	−10.2
α‐5′‐d(TCATAACTGGAT) (ODN‐**3**) β‐5′‐d(AGTATTG1CCTA) (ODN‐**5**)	44	+3	−68	−186	−9.9
α‐5′‐d(TCATAACTGGAT) (ODN‐**3**) β‐5′‐d(AGT1TTG1CCTA) (ODN‐**6**)	46	+5	−70	−193	−10.4
α‐5′‐d(TCATAACTGGAT) (ODN‐**3**) β‐5′‐d(1GT1TTG1CCTA) (ODN‐**7**)	47	+6	−71	−196	−10.7
α‐5′‐d(TCATAACTGGAT) (ODN‐**3**) β‐5′‐d(AGT2TTGACCTA) (ODN‐**8**)	50	+9	−77	−210	−11.5
α‐5′‐d(TCATAACTGGAT) (ODN‐**3**) β‐5’‐d(AGTATTG2CCTA) (ODN‐**9**)	49	+8	−73	−201	−11.0
α‐5′‐d(TCATAACTGGAT) (ODN‐**3**) β‐5′‐d(AGT2TTG2CCTA) (ODN‐**10**)	55	+14	−81	−211	−12.8
α‐5′‐d(TCATAACTGGAT) (ODN‐**3**) β‐5′‐d(2GT2TTG2CCTA) (ODN‐**11**)	57	+16	−82	−220	−13.3
					
**Homochiral Duplexes Antiparallel Strands**					
β‐5′‐d(TAGGTCAATACT) (ODN‐**1**) β‐3′‐d(ATCCAGTTATGA) (ODN‐**2**)	47		−82	−228	−11.0
β‐5′‐d(TAGGTCAATACT) (ODN‐**1**) β‐3′‐d(ATCCAGTT1TGA) (ODN‐**4**)	50	+3	−92	−259	−12.1
β‐5′‐d(TAGGTCAATACT) (ODN‐**1**) β‐3′‐d(ATCC1GTTATGA) (ODN‐**5**)	49	+2	−86	−239	−11.6
β‐5′‐d(TAGGTCAATACT) (ODN‐**1**) β‐3′‐d(ATCC1GTT1TGA) (ODN‐**6**)	51	+4	−89	−248	−12.2
β‐5′‐d(TAGGTCAATACT) (ODN‐**1**) β‐3′‐d(ATCC1GTT1TG1) (ODN‐**7**)	52	+5	−88	−244	−12.4
β‐5′‐d(TAGGTCAATACT) (ODN‐**1**) β‐3′‐d(ATCCAGTT2TGA) (ODN‐**8**)	54	+7	−88	−242	−12.9
β‐5′‐d(TAGGTCAATACT) (ODN‐**1**) β‐3′‐d(ATCC2GTTATGA) (ODN‐**9**)	52	+5	−88	−243	−12.4
β‐5′‐d(TAGGTCAATACT) (ODN‐**1**) β‐3′‐d(ATCC2GTT2TGA) (ODN‐**10**)	58	+11	−91	−247	−14.3
β‐5′‐d(TAGGTCAATACT) (ODN‐**1**) β‐3′‐d(ATCC2GTT2TG2) (ODN‐**11**)	59	+12	−90	−243	−14.4
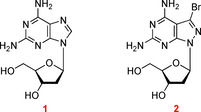					

[a] Measured at 260 nm in 100 mm NaCl, 10 mm MgCl_2_, 10 mm Na‐cacodylate (pH 7.0) with 5 μm+5 μm single‐strand concentration at a heating rate of 1.0 °C min^−1^. *T*_m_ values were calculated from the cooling curves by using the program *Meltwin 3.0*.^[22]^ Δ*T*_m_=*T*_m_ modified duplex−*T*_m_ unmodified duplex. **1** corresponds to purine‐2,6‐diamine 2′‐deoxyribonucleoside. **2** corresponds to 8‐aza‐7‐deaza‐7‐bromopurine‐2,6‐diamine 2′‐deoxyribonucleoside.^[6]^

To shed more light on this matter, *T*_m_ values were listed and thermodynamic data of duplex formation were calculated from shape analysis of the melting profiles (Table 2). Comparison of the thermodynamic data of a set of heterochiral and homochiral duplexes with almost identical *T*_m_ values shows that the enthalpic contribution (Δ*H*) for the formation of base pairs is lower for heterochiral than for homochiral DNA. This is compensated by more favorable entropic terms. It is not clear whether this results from the orientation of strands or from water molecules surrounding single strands and double helices. Water molecules bound to single strands have to be reorganized during duplex formation. This process might be less entropy consuming in the formation of heterochiral duplexes than for homochiral ones.

According to the *T*_m_ values and thermodynamic data, the 7‐bromo substituent of nucleoside **2** is well‐accommodated in the groove of heterochiral DNA, implying that also larger side chains might be accepted without disturbing the heterochiral double helix structure. So, the stabilizing properties of nucleoside **2** seen for homochiral DNA are also observed for heterochiral DNA with an increase of 5–9 °C per base modification.

The strong stabilization induced by **2** points to the formation of a third hydrogen bond in the base pair under participation of the 2‐amino group. Such a strong stabilization was not observed for homochiral DNA containing the non‐brominated analog of nucleoside **2**, so bromination plays the key role.^[6c]^ Clearly, the 2‐amino group becomes more acidic in the presence of a 7‐bromo substituent, which is in accordance with the p*K* values. Consequently, the formation of a third hydrogen bond is promoted. However, also stronger stacking interactions have to be taken in account, and the lipophilic character of the bromo base can stabilize the double helix by expelling water molecules from the grooves. In addition, the single‐crystal X‐ray structure of nucleoside **2** shows conformational characteristics different to nucleoside **1**.^[21]^

### Impact of nucleosides 1 and 2 on the helix structure of heterochiral and homochiral DNA

**Figure 4:**
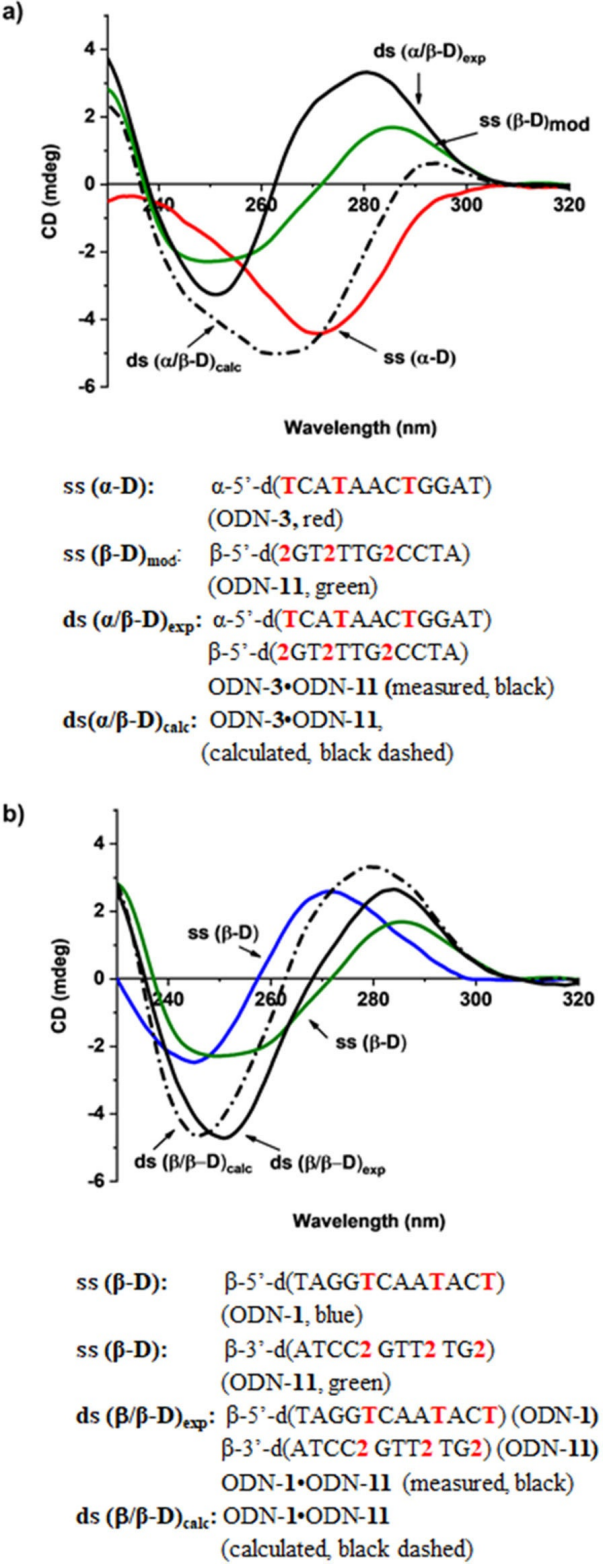
CD‐spectra (a,b) of single‐stranded oligonucleotides and duplexes measured in 100 mm NaCl, 10 mm MgCl_2_, 10 mm Na‐cacodylate, pH 7.0 with 5 μm single strand and 5 μm duplex concentration. The cell path length of the cuvette was 5 mm.

CD spectra are used to study global helical changes of DNA.^[23]^ To this end, CD spectra of oligonucleotide single strands with nucleosides **1** and **2** were measured and compared with those containing dA (Figure 4 and Figure S19 in the Supporting Information). Whereas, the CD spectra of single strands with an increasing number of incorporations of nucleoside **2** show a substantial bathochromic shift of the CD maxima near 270 nm compared with those with dA, oligonucleotides incorporating purine‐2,6‐diamine nucleoside **1** show almost no shift (Figure S18 in the Supporting Information).

Figure 4 shows the situation for heterochiral (Figure 4 a) and homochiral (Figure 4 b) DNA duplexes incorporating the 8‐aza‐7‐deaza‐7‐bromopurine‐2,6‐diamine nucleoside **2**. CD spectra are displayed for (i) complementary single strands, (ii) a measured CD spectrum of the duplex, and (iii) a calculated duplex spectrum generated by the addition of both spectra of the single strands.

From these spectra the following conclusions can be drawn:

(i) The measured CD spectra of heterochiral and homochiral duplexes are both typical for B‐DNA like structures formed by a right‐handed helix with local maxima around 270–280 nm and local minima around 250 nm. However, there is a shoulder in the positive lobe of the heterochiral spectrum (ODN‐**3⋅**ODN‐**11**), which is not found in the curve of the homochiral duplex (ODN‐**1⋅**ODN‐**11**).

(ii) On the contrary to the measured spectrum, the calculated curve of the heterochiral duplex shows a strong negative lobe at 250 nm and a maximum around 270 nm. This is the result of the almost mirror‐like spectrum of the α‐D single strand compared with the β‐D strand.

(iii) From the differences of the measured and calculated CD spectra of the heterochiral duplex, it is apparent that the β‐D strand dictates the helicity of the duplex over the α‐D strand.

(iv) CD spectra of heterochiral and homochiral DNA duplexes modified with the purine‐2,6‐diamine nucleoside **1** (Figure S20 in the Supporting Information) show the same tendencies as described for the corresponding duplexes containing nucleoside **2**.

**Figure 5:**
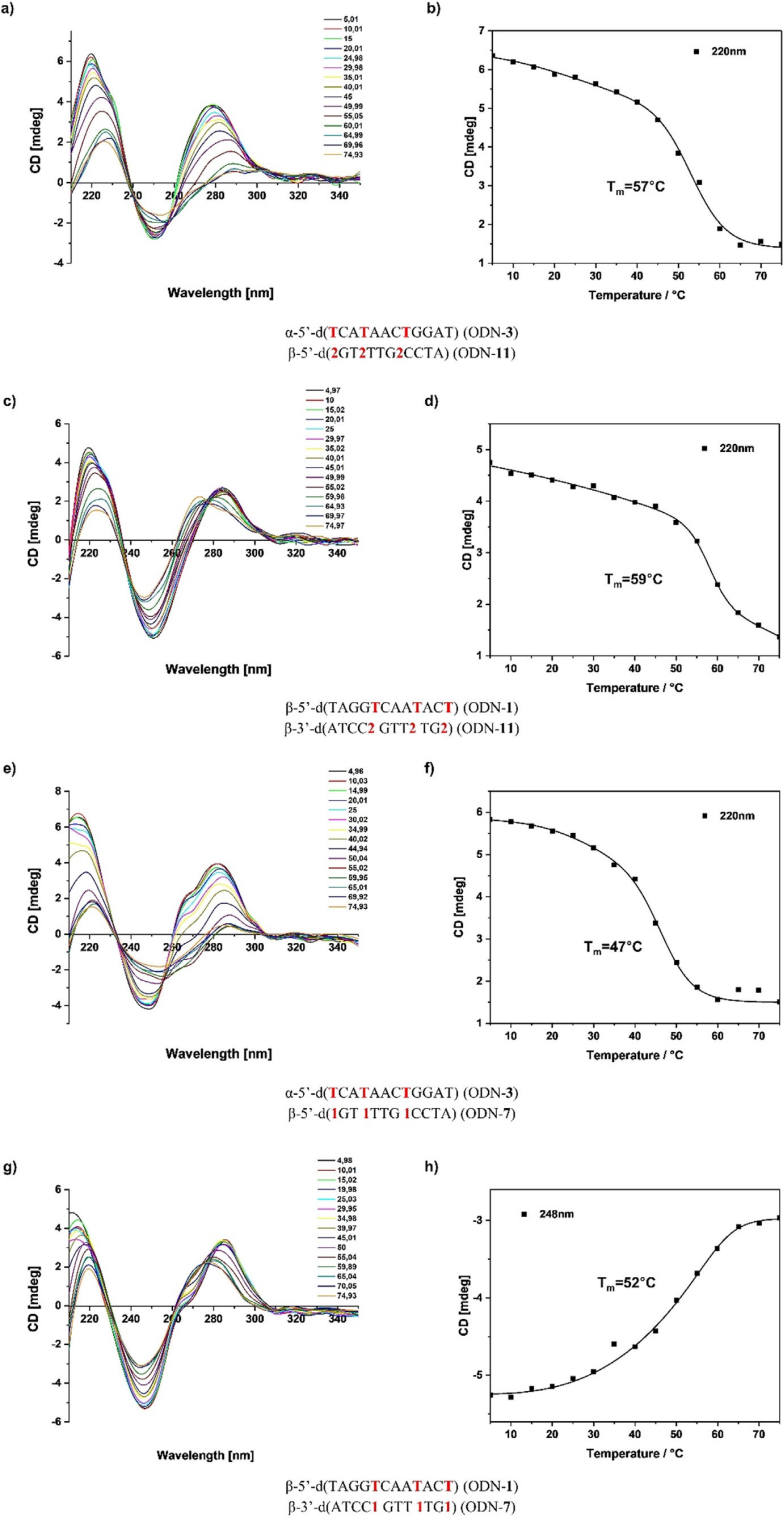
Temperature‐dependent CD spectra of hetero‐ and homochiral duplexes. a) ODN‐**3⋅**ODN‐**11**; c) ODN‐**1⋅**ODN‐**11**; e) ODN‐**3⋅**ODN‐**7**; g) ODN‐**1⋅**ODN‐**7**. CD melting curves obtained from temperature‐dependent CD spectra. b) ODN‐**3⋅**ODN‐**11**; d) ODN‐**1⋅**ODN‐**11**; f) ODN‐**3⋅**ODN‐**7**; h) ODN‐**1⋅**ODN‐**7**. All measurements were performed in 100 mm NaCl, 10 mm MgCl_2_, 10 mm Na‐cacodylate, pH 7.0. The cell path length of the cuvette for the CD spectra was 5 mm.

As conformational changes occurring during melting of DNA cannot be uncovered by UV melting curves, temperature‐dependent CD spectra were used for this purpose. CD spectra of heterochiral and homochiral DNA were measured with three incorporations of nucleosides **1** or **2** (Figure 5). According to Figure 5, the curves at low temperature for both species (heterochiral/homochiral) show the shape of a B‐DNA as reported above. During melting of homochiral duplexes, a strong hypsochromic shift (≈10 nm) of the positive lobe at 285 nm with almost no change of the amplitude was observed. On the contrary, melting of heterochiral duplexes results in a bathochromic shift (10 nm) of the positive lobe accompanied by a strong reduction of the amplitude. Moreover, the shape of both sets of melting curves (homochiral and heterochiral) are different to those reported recently having dA–dT base pairs instead of **2**–dT pairs.^[8]^ In particular, the hypsochromic (homochiral duplexes) and bathochromic (heterochiral duplexes) shifts are more characteristic for the **2**–dT pair. When the CD data at 220 nm were plotted against the ellipticity, *T*_m_ values were obtained that are almost identical to those of the UV melting curves.

**Figure 6:**
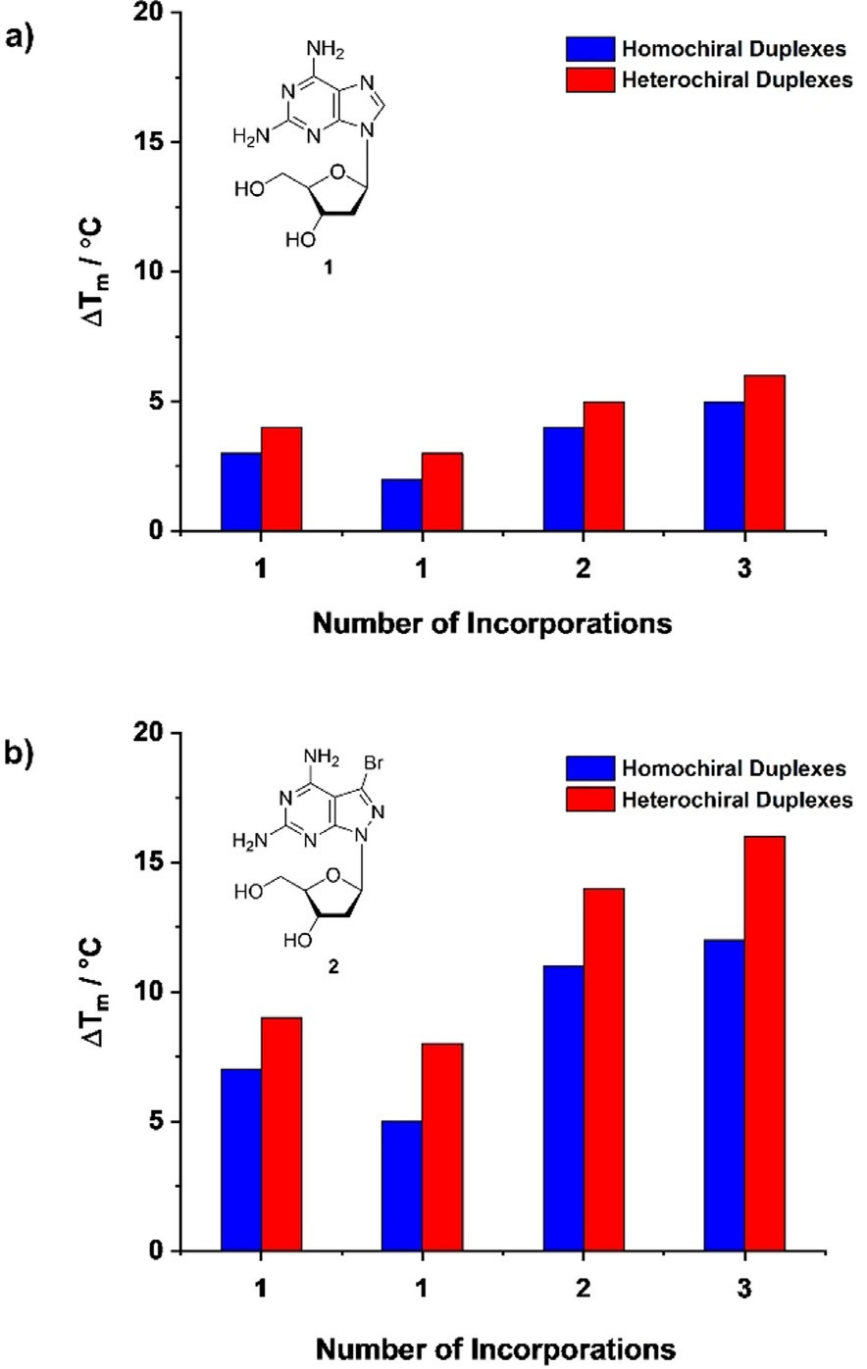
Bar diagrams displaying the *T*_m_ increase of heterochiral (blue) and homochiral (red) duplexes generated by increasing numbers of incorporations of the modified nucleosides **1** (upper part) and **2** (lower part).

For nucleoside **1** as part of homochiral duplexes, a number of publications exist investigating the impact of the 2‐amino group.^[24]^ In homochiral B‐DNA, the 2‐amino group of **1** protrudes in the minor groove of the double helix. This is the same for the amino group of **2**. As a consequence, conformational parameters (propeller‐twist, slide) are altered for DNA duplexes containing purine‐2,6‐diamine tracts. Also, according to the smaller size of the minor groove, steric hindrance by the 2‐amino group has been reported.[^4e^, ^24^] This results in reduced helix flexibility accompanied by an increase of the persistence length of the modified DNA molecule.^[24a]^ The situation can be different for heterochiral DNA as groove size might differ with respect to homochiral DNA. This will influence the impact of the 2‐amino group on the double helix structure, which might result in a decrease of steric hindrance and a better accommodation in the groove. Nevertheless, nucleoside **2** is a much stronger DNA stabilizer for the dA–dT base pair than the naturally occurring **1**; both with a stronger stabilizing effect for heterochiral DNA. The bar diagram in Figure 6 illustrates the situation for 12‐mer duplexes and compares the stabilizing effect of the 8‐aza‐7‐deaza‐7‐bromopurine‐2,6‐diamine nucleoside **2** to the purine‐2,6‐diamine compound **1** in heterochiral and homochiral DNA. It is clear that the stabilization is much higher for **2** replacing dA in dA–dT base pairs than for **1**. So far, compound **2** displays the best stabilizer ability for heterochiral DNA duplexes, which represent an autonomous DNA structure formed by complementary α‐D and β‐D oligonucleotide chains.

## Conclusion

Modified nucleosides represent an important class of molecules useful for the stabilization of nucleic acids. They are beneficial to DNA stability in materials science, are used in biotechnology, and are applied to DNA diagnostics. So far, a number of modified nucleosides have been reported and were incorporated in canonical homochiral DNA.[^1a^, ^3a^, ^3b^] Compared with that, little is known about their stabilizing effect on heterochiral DNA with complementary strands in α‐D and β‐D configuration. Nucleosides **1** and **2**, which carry two amino groups at the 2‐ and 6‐positions, have the capability to form tridentated base pairs with dT and are used to stabilize heterochiral DNA. In detail, the stabilization by nucleosides **1** and **2** was studied on 12‐mer oligonucleotides prepared by solid‐phase synthesis using phosphoramidites **3** and **4**. To this end, a short efficient route for the synthesis of the phosphoramidite **3** was developed. Depurination as easily occurring on purine‐2,6‐diamine nucleosides was not observed. Global helical changes on heterochiral DNA occurring during duplex formation and melting were identified by CD spectra. According to this, the β‐strand always controls the helicity of heterochiral duplexes, thereby forming a B‐type DNA. The increase of duplex stability determined from melting profiles and thermodynamic data indicate that the 8‐aza‐7‐deaza‐7‐bromopurine‐2,6‐diamine nucleoside **2** is an excellent stabilizer for heterochiral DNA (16 °C for three replacements of dA by **2**) whereas the effect of the purine nucleoside **1** is weak. So far, nucleoside **2** was found to be the most efficient structural analog of **1** to stabilize the autonomous DNA pairing system formed by complementary strands in anomeric configuration and strands with parallel orientation.

## Experimental Section

### General methods and materials

Compounds shown in Figure 1. **1**: 9‐(2‐Deoxy‐β‐d‐*erythro*‐pentofuranosyl)‐purine‐2,6‐diamine. **2**: 3‐Bromo‐1‐(2‐deoxy‐β‐d‐*erythro*‐pentofuranosyl)‐1 *H*‐pyrazolo[3,4‐*d*]pyrimidine‐4,6‐diamine. **3**: *N* ^6^‐[(Di‐ *n*‐butylamino)methylidene]‐*N* ^2^‐(phenoxyacetyl)‐9‐[2‐deoxy‐5‐*O*‐(4,4′‐dimethoxytrityl)‐β‐d‐*erythro*‐pentofuranosyl]‐purine‐2,6‐diamine 3′‐(2‐cyanoethyl)‐*N*,*N*‐diisopropylphosphoramidite. **4**: 3‐Bromo‐1‐[2‐deoxy‐5‐*O*‐(4,4′‐dimethoxytrityl)‐β‐d‐*erythro*‐pentofuranosyl]‐*N* ^4^‐[(di‐*n*‐butylamino)methylidene]‐*N* ^6^‐formyl‐1 *H*‐pyrazolo[3,4‐*d*]pyrimidine‐4,6‐diamine 3′‐(2‐cyanoethyl)‐*N*,*N*‐diisopropylphosphoramidite. The phosphoramidite **4** was prepared according to a literature protocol.^[6a]^ All chemicals and solvents were of laboratory grade as obtained from commercial suppliers and were used without further purification. Thin‐layer chromatography (TLC) was performed on TLC aluminium sheets covered with silica gel 60 F254 (0.2 mm). Flash column chromatography (FC): silica gel 60 (40–60 μm) at 0.4 bar. UV spectra were recorded with a UV spectrophotometer: *λ*_max_ (*ϵ*) in nm, *ϵ* in dm^3^ mol^−1^ cm^−1^. NMR spectra were measured at 599.74 MHz for ^1^H, 150.82 MHz for ^13^C, and 121.52 MHz for ^31^P. ^1^H‐^13^C correlated (HMBC, HSQC) NMR spectra were used for the assignment of the ^13^C signals (Table S1 in the Supporting Information). The *J* values are given in Hz; *δ* values in ppm relative to Me_4_Si as internal standard. For NMR spectra recorded in [D_6_]DMSO, the chemical shift of the solvent peak was set to 2.50 ppm for ^1^H NMR and 39.50 ppm for ^13^C NMR spectroscopy. ESI‐TOF mass spectra of nucleosides were recorded with a Micro‐TOF spectrometer. Reversed‐phase HPLC was carried out by using Hitachi 655A‐12 or L6200A intelligent pumps connected to a 655A variable‐wavelength UV monitor, a controller, and an integrator using a 4×250 mm RP‐18 (10 mm) LiChrospher 100 column. The molecular masses of the oligonucleotides were determined by MALDI‐TOF mass spectrometry with a Bruker Autoflex Speed instrument in linear positive mode with 3‐hydroxypicolinic acid (3‐HPA) as a matrix. Thermal melting curves were measured with an Agilent Technologies Cary 100 Bio UV/Vis spectrophotometer equipped with a thermoelectric controller. The temperature was measured continuously in the reference cell with a Pt‐100 resistor, employing a heating rate of 1 °C min^−1^. *T*_m_ values were determined from the melting curves by using the software *Meltwin*, version 3.0.^[22]^ CD spectra were recorded at 25 °C with a JASCO J‐815 spectrometer.

### Oligonucleotide syntheses and characterization

Solid‐phase oligonucleotide syntheses were performed with an ABI 392‐08 synthesizer at 1 μmol scale (trityl‐on mode) employing the phosphoramidites of **1** and **2** as well as standard building blocks, giving an average coupling yield of over 95 %. After cleavage from the solid support, the oligonucleotides were deprotected in 28 % aqueous ammonia at 55 °C for 12 h. The 4,4′‐dimethoxytrityl containing oligonucleotides were purified by reversed‐phase HPLC (RP‐18) with a gradient system at 260 nm: (A) MeCN, (B) 0.1 m (Et_3_NH)OAc (pH 7.0)/MeCN, 95:5; gradient I: 0–3 min 10–15 % A in B, 3–15 min 15–50 % A in B; flow rate 0.7 mL min^−1^. The purified “trityl‐on” oligonucleotides were treated with 2.5 % CHCl_2_COOH/CH_2_Cl_2_ for 2 min at 8 °C to remove the 4,4′‐dimethoxytrityl residues. The detritylated oligomers were further purified by reversed‐phase HPLC with gradient II: 0–20 min 0–20 % A in B; 20–25 min, 20 % A in B; flow rate 0.7 mL min^−1^. The oligonucleotides were desalted on a reversed‐phase column (RP‐18) by using water for the elution of salts, and the oligonucleotides were eluted with H_2_O/MeOH (2:3). The oligonucleotides were lyophilized with a Speed‐Vac evaporator to yield colorless solids, which were frozen at −24 °C. The purity of all oligonucleotides was confirmed by RP‐18 HPLC (Figures S1–S11 in the Supporting Information) and MALDI‐TOF mass spectrometry (Table 1). The extinction coefficients *ϵ*_260_ (H_2_O) of the nucleosides were determined as: dA 15 400, dG 11 700, dT 8800, dC 7300, **1** 8200 (MeOH),^[25]^ **2** 8700^[6a]^ mol^−1^dm^3^ cm^−1^. The extinction coefficients of the oligonucleotides were calculated from the sum of the extinction coefficients of their constituent nucleosides, with a hypochromic change of 20 % for the single strands.

### *N* ^2^‐(Phenoxyacetyl)‐9‐(2‐deoxy‐β‐d‐*erythro*‐pentofuranosyl)‐purine‐2,6‐diamine (5)

Compound **1** (2 g, 7.5 mmol) was dissolved in pyridine (60 mL) at 4 °C and treated with trimethylsilyl chloride (9.8 mL, 77.41 mmol), and the solution was stirred for 1 h at 4 °C. The reaction mixture was diluted with MeCN (70 mL), kept at 4 °C, treated with phenoxyacetyl chloride (3.7 mL, 27.05 mmol), and finally stirred for 40 min at 4 °C. After CH_2_Cl_2_ (200 mL) was added, the mixture was poured into H_2_O (200 mL). The organic phase was separated, dried over Na_2_SO_4_ and evaporated, and the residue was co‐evaporated with toluene (3×25 mL). The resulting foam was dissolved in MeOH/THF/28 % aq. NH_3_ solution (1:1:1, 150 mL). After the solution was stirred for 1 h at room temperature, the solvent was evaporated, and the remaining residue was co‐evaporated with toluene (3×30 mL) and finally subjected to FC (silica gel column, 12×4 cm, CH_2_Cl_2_/MeOH 95:5→85:15) to afford compound **5** (1.63 mg, 54 %) as a white solid. *R*_f_=0.25 (CH_2_Cl_2_/MeOH, 90:10); ^1^H NMR (600 MHz, [D_6_]DMSO, 26 °C): *δ*=2.25 (ddd, *J=*3.1, 6.1, 13.1 Hz, 1 H; C2′‐H), 2.67 (ddd, *J=*5.8, 7.8, 13.4 Hz, 1 H; C2′′‐H), 3.55 (ddt, *J=*5.1, 11.7, 43.9 Hz, 2 H; 2×C5′‐H), 3.84 (td, *J=*2.7, 4.7 Hz, 1 H; C4′‐H), 4.37–4.42 (m, 1 H; C3′‐H), 4.92 (t, *J=*5.5 Hz, 1 H; C5′‐OH), 5.02 (s, 2 H; OCH_2_), 5.29 (d, *J=*3.9 Hz, 1 H; C3′‐OH), 6.26–6.29 (m, 1 H; C1′‐H), 6.91–6.97 (m, 3 H; Ar‐H), 7.27–7.38 (m, 4 H; 2×Ar‐H, 6‐NH_2_), 8.25 (s, 1 H; C8‐H), 10.03 ppm (s, 1 H; NH); ^13^C NMR (151 MHz, [D_6_]DMSO, 26 °C): *δ*=39.1 (C‐2′), 61.7 (C‐5′), 67.4 (OCH_2_), 70.8 (C‐3′), 82.9 (C‐1′), 87.7 (C‐4′), 114.5 (arom. C), 116.0 (C‐5), 120.8 (arom. C), 129.4 (arom. C), 138.5(C‐8), 150.0 (C‐4), 152.4 (C‐2), 156.0 (C‐6), 158.0 ppm (C=O); UV/Vis (MeOH): *λ*_max_ (*ϵ*)=270 (18 000), 225 nm (30 500 mol^−1^ dm^3^ cm^−1^); HRMS (ESI‐TOF): *m*/*z* calcd for C_18_H_20_N_6_NaO_5_^+^: 423.1387 [*M*+Na]^+^; found: 423.1377.

### *N* ^6^‐[(Di‐*n*‐butylamino)methylidene]‐*N* ^2^‐(phenoxyacetyl)‐9‐[2‐deoxy‐β‐d‐*erythro*‐pentofuranosyl]‐purine‐2,6‐diamine (6) and *N* ^6^‐[(di‐*n*‐butylamino)methylidene]‐9‐[2‐deoxy‐β‐d‐*erythro*‐pentofuranosyl]‐purine‐2,6‐diamine (7)

Compound **5** (100 mg, 0.25 mmol) was dissolved in MeOH (6 mL). Then, *N*,*N*‐dibutylaminomethylidene dimethylacetal (162 mg, 0.8 mmol) was added and the solution was stirred at 40 °C for 2 h. After depletion of the starting material, the solvent was removed, and the remaining residue was adsorbed on silica gel and subjected to FC (silica gel column, 12×4 cm, CH_2_Cl_2_/MeOH 97:3→90:10). From the faster migrating zone, compound **6** (62 mg, 46 %) was obtained as a colorless solid and from the slower migrating zone, compound **7** was obtained (51 mg, 50 %).

**Compound 6**: *R*_f_=0.5 (CH_2_Cl_2_/MeOH, 90:10); ^1^H NMR (400 MHz, [D_6_]DMSO, 26 °C): *δ*=0.91 (dt, *J=*7.3, 14.8 Hz, 6 H; 2×CH_3_), 1.30 (ddq, *J=*7.5, 14.7, 17.0 Hz, 4 H; 2×CH_2_), 1.60 (ddt, *J=*7.3, 12.5, 16.5 Hz, 4 H; 2×CH_2_), 2.27 (ddd, *J=*3.2, 6.2, 13.1 Hz, 1 H; C2′‐H), 2.69 (ddd, *J=*5.8, 7.6, 13.2 Hz, 1 H; C2′′‐H), 3.41 (t, *J=*7.1 Hz, 2 H; 2×C5′‐H), 3.48–3.65 (m, 4 H; 2×NCH_2_), 3.85 (td, *J=*2.8, 4.7 Hz, 1 H; C4′‐H), 4.41 (dt, *J=*3.2, 6.3 Hz, 1 H; C3′‐H), 4.90 (t, *J=*5.5 Hz, 1 H; C5′‐OH), 5.03 (s, 2 H; OCH_2_), 5.30 (d, *J=*4.0 Hz, 1 H; C3′‐OH), 6.33 (dd, *J=*6.1, 7.5 Hz, 1 H; C1′‐H), 6.89–6.98 (m, 3 H; Ar‐H), 7.29 (dd, *J=*7.1, 9.0 Hz, 2 H; Ar‐H), 8.35 (s, 1 H; C8‐H), 8.94 (s, 1 H; N=CH), 10.30 ppm (s, 1 H; NH); ^13^C NMR (101 MHz, [D_6_]DMSO, 26 °C): *δ*=13.5 (CH_3_), 13.7 (CH_3_), 19.1 (CH_2_), 19.6 (CH_2_), 28.6 (CH_2_), 30.4 (CH_2_), 39.0 (C‐2′), 44.5 (NCH_2_), 51.0 (NCH_2_), 61.6 (C‐5′), 67.3 (OCH_2_), 70.7 (C‐3′), 83.0 (C‐1′), 87.7 (C‐4′), 114.4 (arom. C), 120.8 (arom. C), 122.3 (C‐5), 129.4 (arom. C), 140.3 (C‐8), 151.9 (C‐4), 152.1 (C‐2), 157.9 (C=O), 158.4 (N=CH), 159.7 ppm (C‐6); UV/Vis (MeOH): *λ*_max_ (*ϵ*)=320 (31 000), 260 nm (24 000 mol^−1^ dm^3^ cm^−1^); HRMS (ESI‐TOF): *m*/*z* calcd for C_27_H_37_N_7_O_5_: 540.2929 [*M*+H]^+^; found: 540.2927.

**Compound 7**: *R*_f_=0.37 (CH_2_Cl_2_/MeOH, 90:10); ^1^H NMR (400 MHz, [D_6_]DMSO, 26 °C): *δ*=0.92 (td, *J=*2.1, 7.3 Hz, 6 H; 2×CH_3_), 1.22–1.37 (m, 4 H; 2×CH_2_), 1.51–1.63 (m, 4 H; 2×CH_2_), 2.19 (ddd, *J=*2.9, 6.1, 13.0 Hz, 1 H; C2′‐H), 2.58 (ddd, *J=*5.7, 8.1, 13.4 Hz, 1 H; C2′′‐H), 3.36 (t, *J=*7.2 Hz, 2 H; NCH_2_), 3.46–3.63 (m, 4 H, 2×C5′‐H; NCH_2_), 3.83 (td, *J=*2.5, 4.4 Hz, 1 H; C4′‐H), 4.35 (dq, *J=*2.9, 6.0 Hz, 1 H; C3′‐H), 5.09 (dd, *J=*5.2, 6.0 Hz, 1 H; C5′‐OH), 5.25 (d, *J=*4.0 Hz, 1 H; C3′‐OH), 5.96 (s, 2 H; 2‐NH_2_), 6.21 (dd, *J=*6.0, 8.0 Hz, 1 H; C1′‐H), 8.01 (s, 1 H; C8‐H), 8.79 ppm (s, 1 H; N=CH); ^13^C NMR (101 MHz, [D_6_]DMSO, 26 °C): *δ*=13.5 (CH_3_), 13.7 (CH_3_), 19.1 (CH_2_), 19.6 (CH_2_), 28.6 (CH_2_), 30.5 (CH_2_), 39.1 (C‐2′), 44.2 (NCH_2_), 50.8 (NCH_2_), 61.8 (C‐5′), 70.8 (C‐3′), 82.7 (C‐1′), 87.5 (C‐4′), 119.3 (C‐5), 137.3 (C‐8), 153.1 (C‐4), 157.6 (N=CH), 159.9 (C‐2), 160.0 ppm (C‐6); UV/Vis (MeOH): *λ*_max_ (*ϵ*)=325 (19 200), 278 (13 200), 252 nm (13 200 mol^−1^ dm^3^ cm^−1^); HRMS (ESI‐TOF): *m*/*z* calcd for C_19_H_31_N_7_O_3_^+^: 406.2561 [*M*+H]^+^; found: 406.2559.

### *N* ^6^‐[(Di‐*n*‐butylamino)methylidene]‐*N* ^2^‐(phenoxyacetyl)‐9‐[2‐deoxy‐β‐d‐*erythro*‐pentofuranosyl]‐purine‐2,6‐diamine (6)

Compound **5** (1 g, 2.5 mmol) was dissolved in DMF (5 mL). Then, *N*,*N*‐dibutylaminomethylidene dimethylacetal (1.62 g, 8 mmol) was added and the solution was stirred at room temperature for 2 h. After depletion of the starting material, the solvent was removed under reduced pressure at 32 °C, and the remaining residue was adsorbed on silica gel and subjected to FC (CH_2_Cl_2_/MeOH 97:3→90:10), affording compound **6** (832 mg, 62 %) as a colorless solid. Analytical data are identical to that described above.

### *N* ^6^‐[(Di‐*n*‐butylamino)methylidene]‐*N* ^2^‐(phenoxyacetyl)‐9‐[2‐deoxy‐5‐*O*‐(4,4′‐dimethoxytrityl)‐β‐d‐*erythro*‐pentofuranosyl]‐purine‐2,6‐diamine (8)

Compound **6** (500 mg, 0.93 mmol) was dried by repeated co‐evaporation with dry pyridine (3×10 mL) and then dissolved in dry pyridine (10 mL). To this solution, 4,4′‐dimethoxytrityl chloride (470 mg, 1.4 mmol) was added. The mixture was stirred at room temperature for 2 h until the starting material was completely consumed (TLC monitoring). Then, the reaction mixture was diluted with CH_2_Cl_2_ (25 mL) and washed with 5 % aq. NaHCO_3_ solution (20 mL) and water (20 mL). The organic layer was dried over Na_2_SO_4_, the solvent was evaporated under reduced pressure, and the residue was subjected to FC (silica gel, CH_2_Cl_2_/acetone, 90:10→80:20). Evaporation of the main zone afforded **8** (510 mg, 65 %) as a pale‐yellow foam. *R*_f_=0.5 (CH_2_Cl_2_/acetone, 80:20); ^1^H NMR (400 MHz, [D_6_]DMSO, 26 °C): *δ*=0.91 (dt, *J=*7.4, 16.2 Hz, 6 H; 2×CH_3_), 1.25–1.37 (m, 4 H, 2×CH_2_), 1.54–1.67 (m, 4 H, 2×CH_2_), 2.34 (ddd, *J=*5.0, 6.9, 13.3 Hz, 1 H; C2′‐H), 2.85 (dt, *J=*6.3, 12.9 Hz, 1 H, C2′′‐H), 3.09 (dd, *J=*3.3, 10.3 Hz, 1 H; C5′‐H), 3.24 (dd, *J=*6.8, 10.4 Hz, 1 H; C5′′‐H), 3.41 (t, *J=*7.1 Hz, 2 H; NCH_2_), 3.58 (t, *J=*7.5 Hz, 2 H; NCH_2_), 3.69 (d, *J=*4.1 Hz, 6 H; 2×OCH_3_), 3.96 (dt, *J=*3.9, 7.0 Hz, 1 H; C4′‐H), 4.47 (t, *J=*5.3 Hz, 1 H; C3′‐H ), 5.01 (d, *J=*3.4 Hz, 2 H; OCH_2_), 5.31 (d, *J=*4.4 Hz, 1 H; C3′‐OH), 6.37 (t, *J=*6.4 Hz, 1 H; C1′‐H), 6.69–6.80 (m, 4 H; Ar‐H), 6.89–6.99 (m, 3 H; Ar‐H), 7.11–7.23 (m, 7 H; Ar‐H), 7.24–7.33 (m, 4 H; Ar‐H), 8.24 (s, 1 H; C8‐H), 8.93 (s, 1 H; N=CH), 10.28 ppm (s, 1 H; NH); ^13^C NMR (101 MHz, [D_6_]DMSO, 26 °C): *δ*=13.5 (CH_3_), 13.7 (CH_3_), 19.1 (CH_2_), 19.6 (CH_2_), 28.6 (CH_2_), 30.4 (CH_2_), 38.8 (C‐2′), 44.5 (NCH_2_), 51.1 (NCH_2_), 54.9 (OCH_3_), 64.3 (C‐5′), 67.3 (OCH_2_), 70.6 (C‐3′), 83.1 (C‐1′), 85.3 (C_quat_), 86.0 (C‐4′), 112.9 (arom. C), 113.0 (arom. C), 114.4 (arom. C), 120.8 (arom. C), 122.5 (C‐5), 126.4 (arom. C), 127.6 (arom. C), 129.4 (arom. C), 129.5 (arom. C), 129.7 (arom. C), 135.3 (arom. C), 135.5 (arom. C), 140.3 (C‐8), 144.9 (arom. C), 151.8 (C‐4), 152.1 (C‐2), 157.9, 158.0 (C=O), 158.4 (N=CH), 159.8 ppm (C‐6); UV/Vis (MeOH): *λ*_max_ (*ϵ*)=321 (26 700), 260 (24 400), 232 nm (31 100 mol^−1^ dm^3^ cm^−1^); HRMS (ESI‐TOF): *m*/*z* calcd for C_48_H_55_N_7_NaO_7_^+^: 864.4055 [*M*+Na]^+^; found: 864.4055.

### *N* ^6^‐[(Di‐*n*‐butylamino)methylidene]‐*N* ^2^‐(phenoxyacetyl)‐9‐[2‐deoxy‐5‐*O*‐(4,4′‐dimethoxytrityl)‐β‐d‐*erythro*‐pentofuranosyl]‐purine‐2,6‐diamine 3′‐(2‐cyanoethyl)‐*N*,*N*‐diisopropylphosphoramidite (3)

To a solution of compound **8** (250 mg, 0.3 mmol) in dry CH_2_Cl_2_ (4 mL) were added (*i*Pr)_2_NEt (77 mg, 101 μL, 0.6 mmol) and 2‐cyanoethyl diisopropylphosphoramidochloridite (105 mg, 100 μL, 0.45 mmol). Then, the reaction mixture was stirred for 5 min at room temperature. After completion of the reaction (TLC monitoring), the reaction mixture was diluted with CH_2_Cl_2_ (15 mL) and poured into 5 % NaHCO_3_ solution (10 mL). The organic layer was separated, dried over Na_2_SO_4_, and the solvent was evaporated. The residual foam was applied to FC (silica gel, CH_2_Cl_2_/acetone, 90:10). Evaporation of the main zone afforded **3** (211 mg, 68 %) as a colorless foam. *R*_f_=0.5 (CH_2_Cl_2_/acetone 80:20); ^31^P NMR (121 MHz, CDCl_3_, 20 °C): *δ*=148.73, 148.82 ppm; HRMS (ESI‐TOF): *m*/*z* calcd for C_57_H_72_N_9_NaO_8_P^+^: 1064.5134 [*M*+Na]^+^; found: 1064.5132.

## Conflict of interest

The authors declare no conflict of interest.

## Supporting information

As a service to our authors and readers, this journal provides supporting information supplied by the authors. Such materials are peer reviewed and may be re‐organized for online delivery, but are not copy‐edited or typeset. Technical support issues arising from supporting information (other than missing files) should be addressed to the authors.

Supplementary
