# Supplementary material for: The 2‐Amino Group of 8‐Aza‐7‐deaza‐7‐bromopurine‐2,6‐diamine and Purine‐2,6‐diamine as Stabilizer for the Adenine–Thymine Base Pair in Heterochiral DNA with Strands in Anomeric Configuration
Source: Chemistry. 2020 Dec 21;27(6):2093–103. doi: 10.1002/chem.202004221 (PMC7898646; doi:10.1002/chem.202004221)
Supplement: Supplementary file 1 — Supplementary [file CHEM-27-2093-s001.pdf]

Chemistry–A European Journal

Supporting Information

The 2-Amino Group of 8-Aza-7-deaza-7-bromopurine-2,6-diamine and Purine-2,6-diamine as Stabilizer for the Adenine–Thymine Base Pair in Heterochiral DNA with Strands in Anomeric Configuration

Yingying Chai,[a, c] Dasharath Kondhare,[a] Aigui Zhang,[a] Peter Leonard,[a] and Frank Seela*[a, b]

Table of Contents

Table S1. ^{13}C NMR chemical shifts of purine-2,6-diamine nucleosides S2

Figures S1-11. Reversed-phase HPLC profiles of purified oligonucleotides S2-6

Figures S12-13. Melting profiles of heterochiral and homochiral duplexes containing one incorporation of **1** or **2** S7-8

Figure S14. Melting profiles of heterochiral and homochiral duplexes containing two incorporations of **1** or **2** S9

Figure S15. Melting profiles of heterochiral and homochiral duplexes containing three incorporations of **1** or **2** S10

Figure S16. pK_a Determination of nucleoside **1** and **2** by UV S11

Figure S17. UV-spectra of dA, **1** and **2** S12

Figure S18. CD-spectra of single-stranded oligonucleotides S13

Figure S19. CD-spectra of heterochiral and homochiral DNA duplexes S14-15

Figure S20. CD-spectra of heterochiral/homochiral duplexes (calculated and experimental) incorporating **1** or **2** S15

Figure S21-S46. NMR spectra S16-28

Table S1. ^{13}C NMR chemical shifts of purine-2,6-diamine nucleosides^{a}

	C2^{b}	C4^{b}	C5^{b}	C6^{b}	C8^{b}	C=O	N=CH/CH_2/CH_3/OCH_3/qC	C1'	C2'	C3'	C4'	C5'
1[1]	156.1	151.2	113.5	160.0	135.7	--	--	83.1	39.4	71.0	87.6	61.9
**5**	152.4	150.0	116.0	156.0	138.5	158.0	67.4	82.9	39.1	70.8	87.7	61.7
**6**	152.1	151.9	122.3	159.7	140.3	157.9	158.4/67.3/51.0/44.5/30.4/ 28.6/19.6/19.1/13.7/13.5	83.0	39.0	70.7	87.7	61.6
**7**	159.9	153.1	119.3	160.0	137.3	--	157.6/50.8/44.2/30.5/28.6/ 19.6/19.1/13.7/13.5	82.7	39.1	70.8	87.5	61.8
**8**	152.1	151.8	122.5	159.8	140.3	157.95 158.00	158.4/67.3/44.5/51.1/28.6/ 30.4/19.6/19.1/13.7/13.5/ 54.9/85.3	83.1	38.8	70.6	86.0	64.3

^{a} Measured in DMSO-d_6 at 298 K. ^{b} Purine numbering.

[1] M. J. Robins, R. Zou, F. Hansske, S. F. Wnuk, *Can. J. Chem*. **1997**, *75*, 762-767.

Reversed-phase (RP-18) HPLC profiles of oligonucleotides

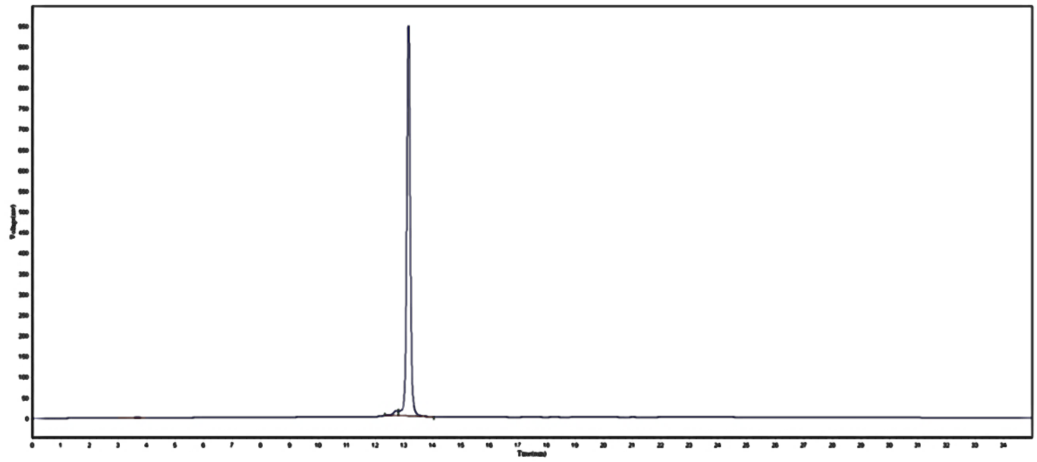

Figure S1. β-5'-d(TAGGTCAATACT) (ODN-**1**)

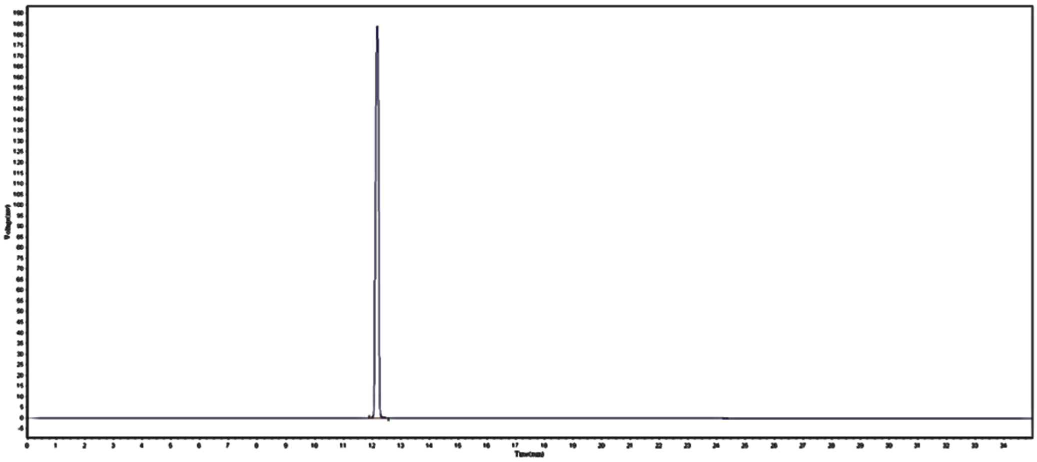

Figure S2. β-5'-d(AGTATTGACCTA) (ODN-**2**)

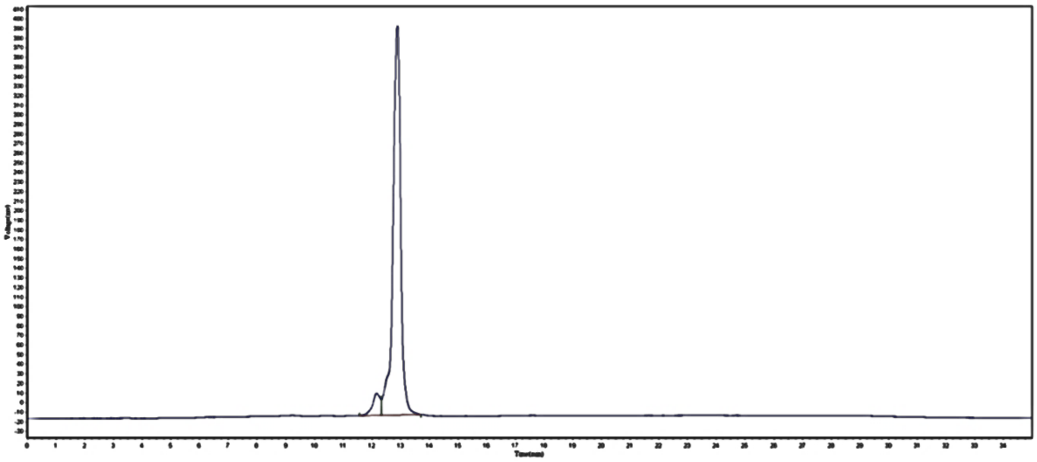

Figure S3. α-5'-d(TCATAACTGGAT) (ODN-**3**)

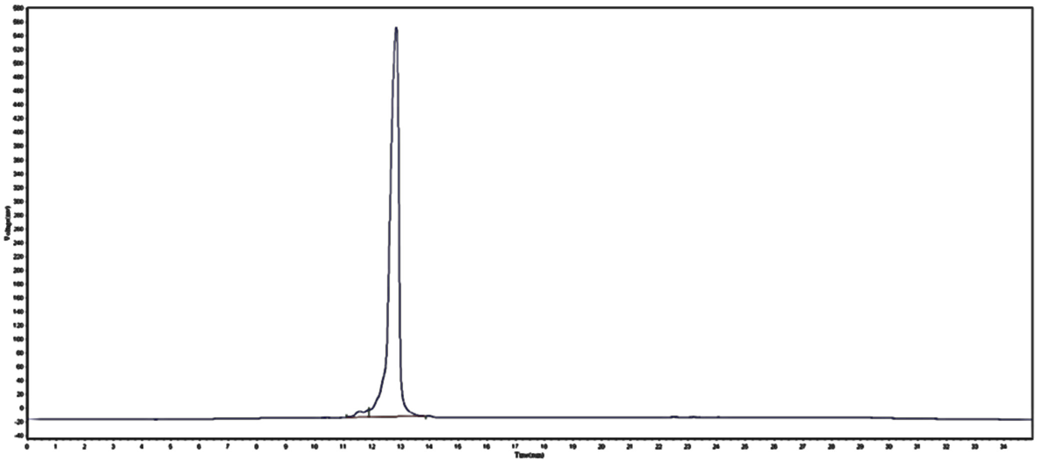

Figure S4. β-5'-d(AGT**1**TTGACCTA) (ODN-**4**)

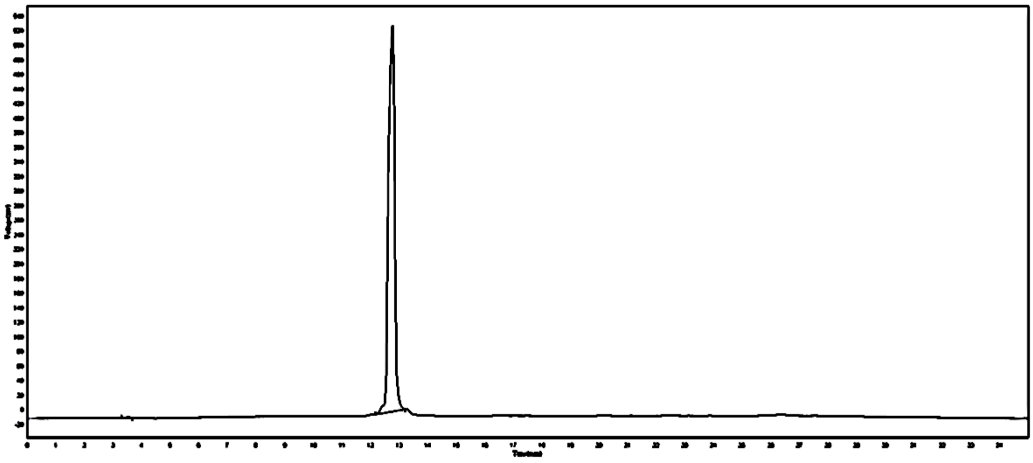

Figure S5. β-5’-d(AGTATTG**1**CCTA) (ODN-**5**)

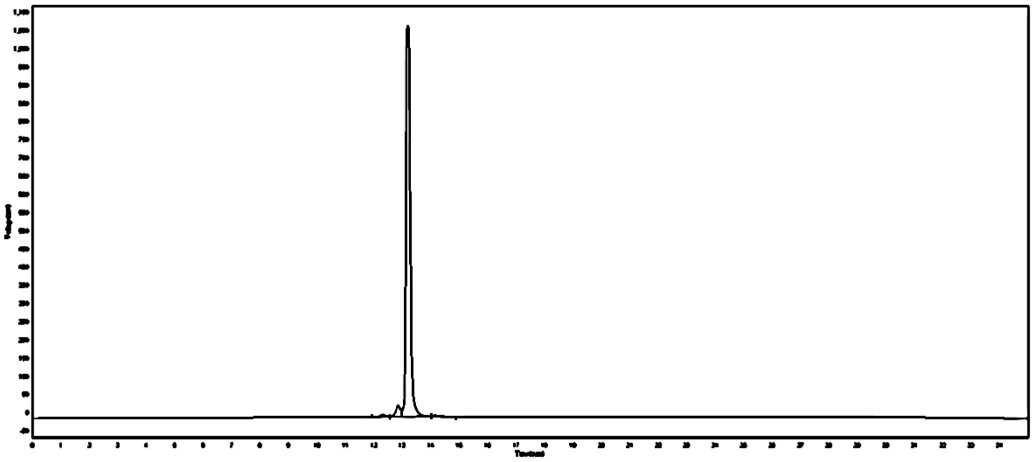

Figure S6. β-5’-d(AGT**1**TTG**1**CCTA) (ODN-**6**)

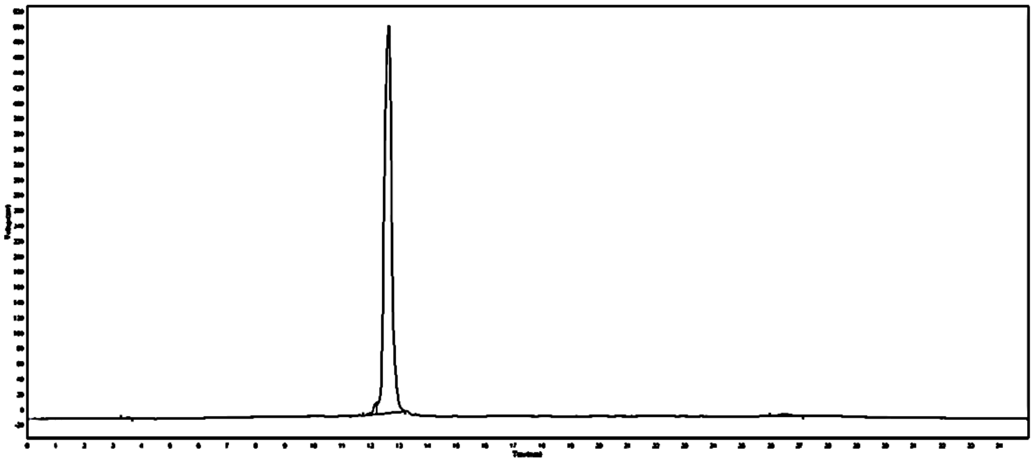

Figure S7. β-5’-d(**1**GT**1**TTG**1**CCTA) (ODN-**7**)

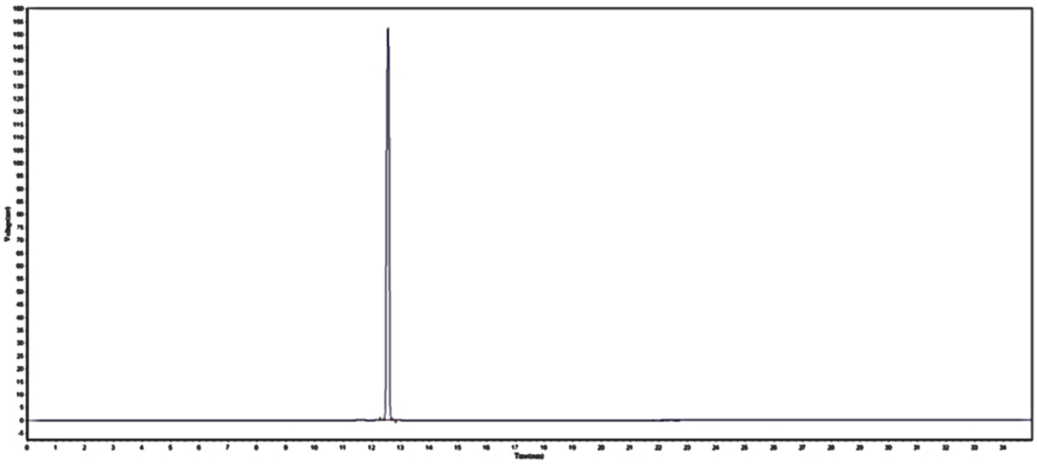

Figure S8. β-5’-d(AGT**2**TTGACCTA) (ODN-**8**)

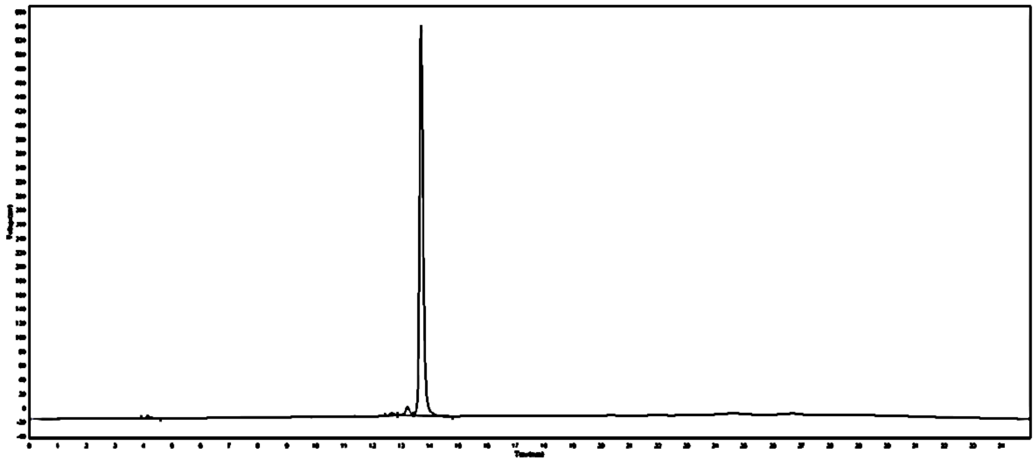

Figure S9. β-5’-d(AGTATTG**2**CCTA) (ODN-**9**)

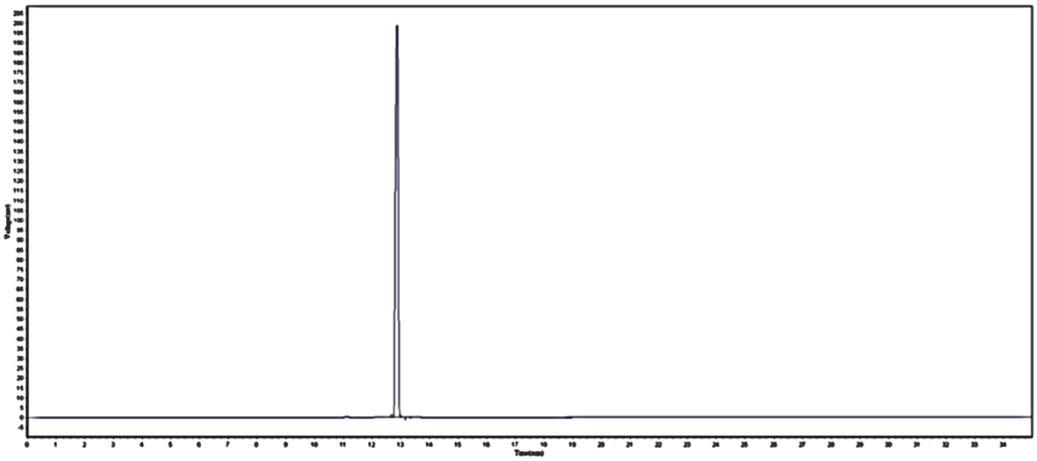

Figure S10. β-5’-d(AGT**2**TTG**2**CCTA) (ODN-**10**)

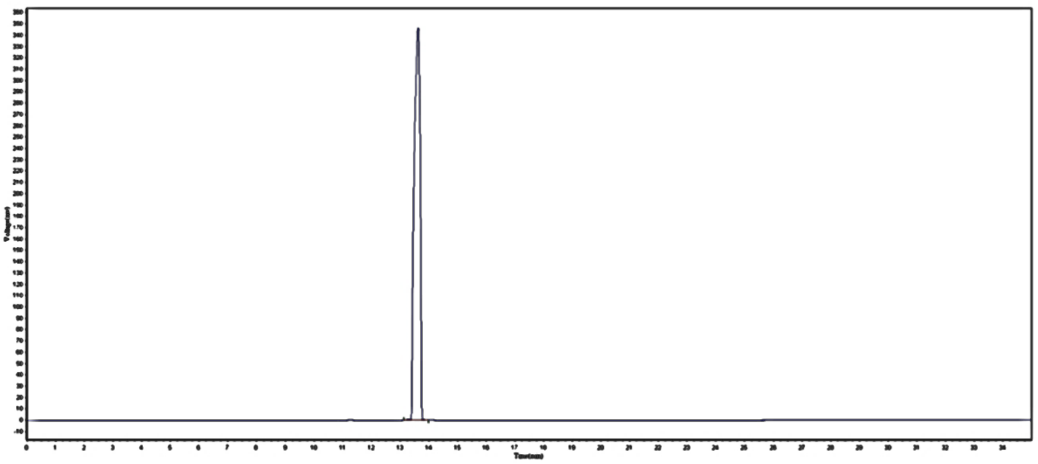

Figure S11. β-5'-d(**2**GT**2**TTG**2**CCTA) (ODN-**11**)

Figures S1-11. Reversed-phase (RP-18) HPLC elution profiles of purified oligonucleotides monitored at 260 nm. X-axis refers to retention time (min); Y-axis refers to UV absorbance at 260 nm, measured in mV. For elution, the following system was used: (A) MeCN, (B) 0.1 M $(Et_3NH)OAc$ (pH 7.0)/MeCN, 95:5; gradient: 0-20 min 0-20% A in B; 20-25 min, 20% A in B; flow rate 0.7 mL/min.

Melting profiles of heterochiral (α/β) and homochiral (β/β) oligonucleotide duplexes containing (α/β)-dT opposite to one incorporation of 1 or 2

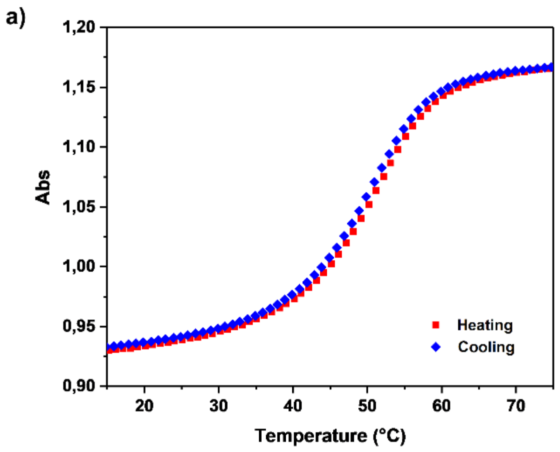

α-5'-d(TCA**T**AACTGGAT) (ODN-**3**)
β-5'-d(AGT**2**TTGACCTA) (ODN-**8**)

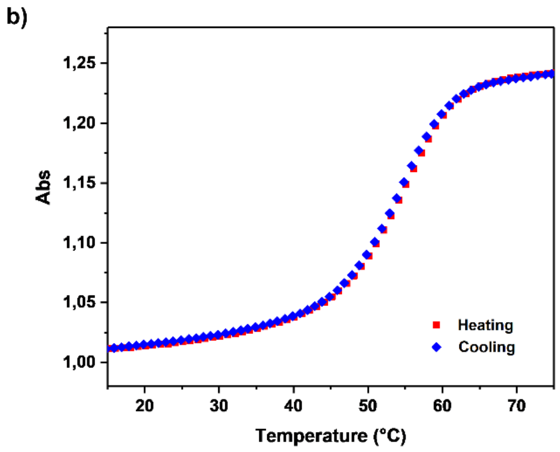

β-5'-d(TAGGTCAA**T**ACT) (ODN-**1**)
β-3'-d(ATCCAGTT**2**TGA) (ODN-**8**)

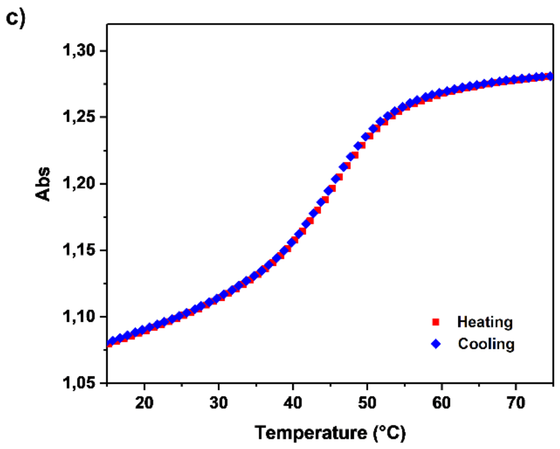

α-5'-d(TCA**T**AACTGGAT) (ODN-**3**)
β-5'-d(AGT**1**TTGACCTA) (ODN-**4**)

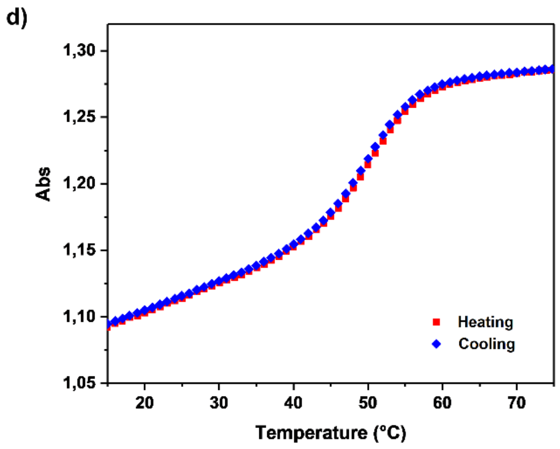

β-5'-d(TAGGTCAA**T**ACT) (ODN-**1**)
β-3'-d(ATCCAGTT**1**TGA) (ODN-**4**)

Figure S12. Thermal denaturation curves of duplexes a) ODN-**3**•ODN-**8**; b) ODN-**1**•ODN-**8**; c) ODN-**3**•ODN-**4**; d) ODN-**1**•ODN-**4** measured with 5 µM + 5 µM single-strand concentration in 100 mM NaCl, 10 mM $MgCl_2$, 10 mM Na-cacodylate buffer (pH = 7.0) at 260 nm.

Melting profiles of heterochiral (α/β) and homochiral (β/β) oligonucleotide duplexes containing (α/β)-dT opposite to one incorporation of 1 or 2

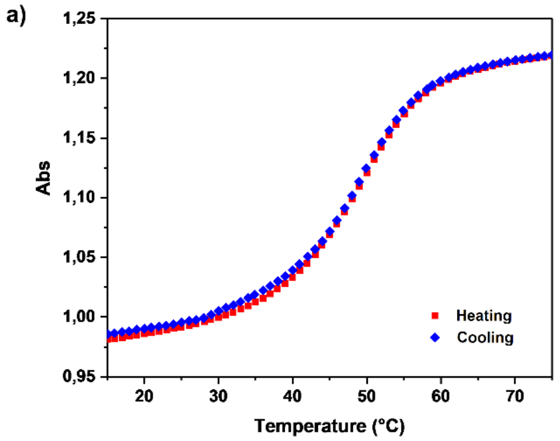

α-5'-d(TCATAACTGGAT) (ODN-**3**)
β-5'-d(AGTATTG2CCTA) (ODN-**9**)

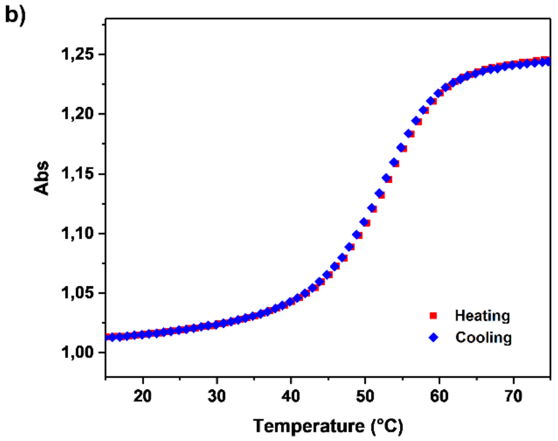

β-5'-d(TAGGTCAATACT) (ODN-**1**)
β-3'-d(ATCC2GTTATGA) (ODN-**9**)

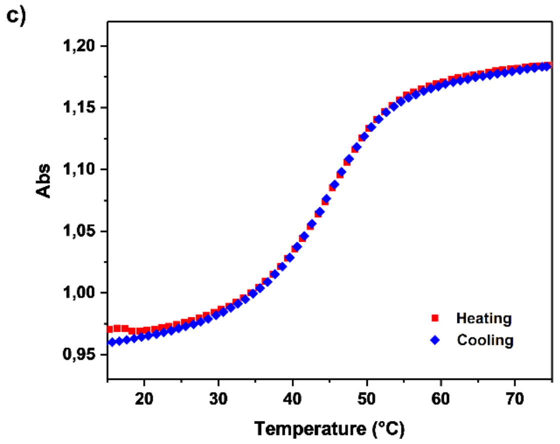

α-5'-d(TCATAACTGGAT) (ODN-**3**)
β-5'-d(AGTATTG1CCTA) (ODN-**5**)

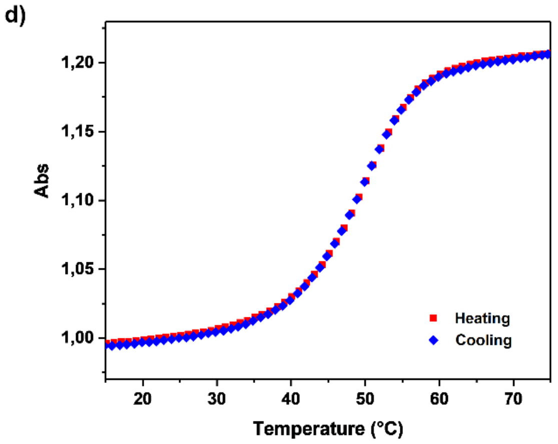

β-5'-d(TAGGTCAATACT) (ODN-**1**)
β-3'-d(ATCC1GTTATGA) (ODN-**5**)

Figure S13. Thermal denaturation curves of duplexes a) ODN-**3**•ODN-**9**; b) ODN-**1**•ODN-**9**; c) ODN-**3**•ODN-**5**; d) ODN-**1**•ODN-**5** measured with 5 µM + 5 µM single-strand concentration in 100 mM NaCl, 10 mM $MgCl_2$, 10 mM Na-cacodylate buffer (pH = 7.0) at 260 nm.

Melting profiles of heterochiral (α/β) and homochiral (β/β) oligonucleotide duplexes containing (α/β)-dT opposite to two incorporations of 1 or 2

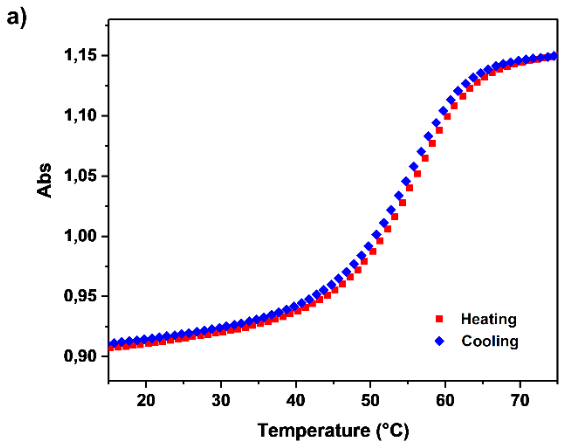

α-5’-d(TCATAACTGGAT) (ODN-**3**)
β-5’-d(AGT2TTG2CCTA) (ODN-**10**)

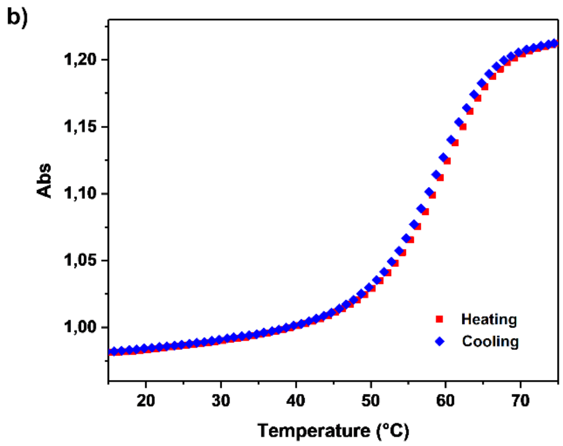

β-5’-d(TAGGTCAATACT) (ODN-**1**)
β-3’-d(ATCC2GTT2TGA) (ODN-**10**)

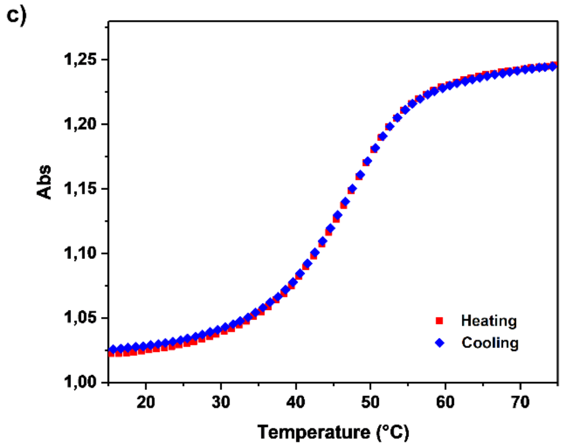

α-5’-d(TCATAACTGGAT) (ODN-**3**)
β-5’-d(AGT1TTG1CCTA) (ODN-**6**)

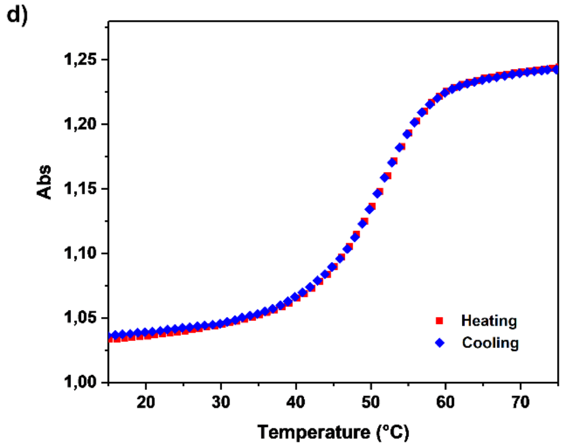

β-5’-d(TAGGTCAATACT) (ODN-**1**)
β-3’-d(ATCC1GTT1TGA) (ODN-**6**)

Figure S14. Thermal denaturation curves of duplexes a) ODN-**3**•ODN-**10**; b) ODN-**1**•ODN-**10**; c) ODN-**3**•ODN-**6**; d) ODN-**1**•ODN-**6** measured with 5 µM + 5 µM single-strand concentration in 100 mM NaCl, 10 mM $MgCl_2$, 10 mM Na-cacodylate buffer (pH = 7.0) at 260 nm.

Melting profiles of heterochiral (α/β) and homochiral (β/β) oligonucleotide duplexes containing (α/β)-dT opposite to three incorporations of 1 or 2

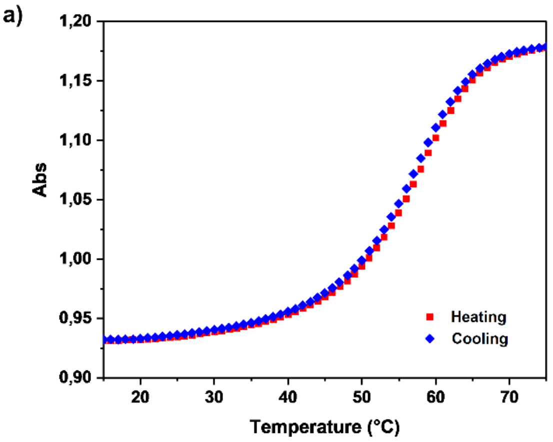

α-5'-d(TCATAACTGGAT) (ODN-**3**)
β-5'-d(2GT2TTG2CCTA) (ODN-**11**)

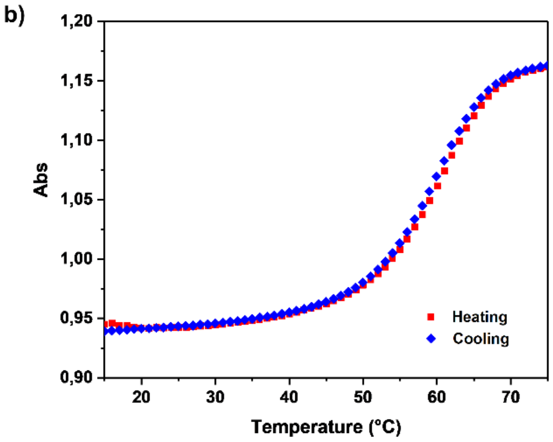

β-5'-d(TAGGTCAATACT) (ODN-**1**)
β-3'-d(ATCC2GTT2TG2) (ODN-**11**)

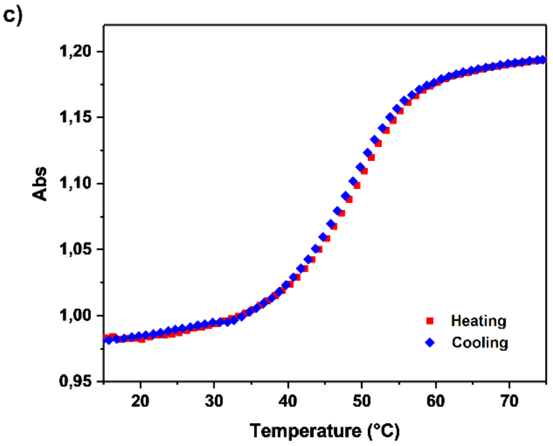

α-5'-d(TCATAACTGGAT) (ODN-**3**)
β-5'-d(1GT1TTG1CCTA) (ODN-**7**)

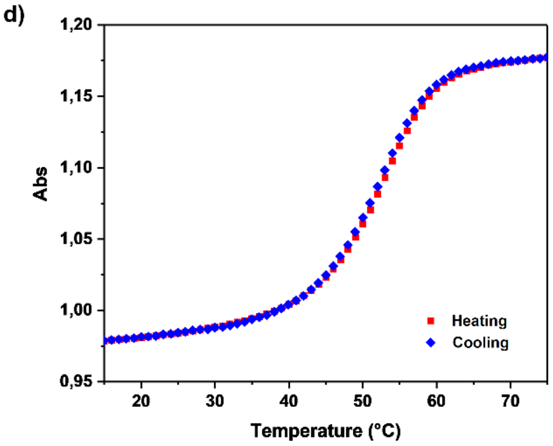

β-5'-d(TAGGTCAATACT) (ODN-**1**)
β-3'-d(ATCC1GTT1TG1) (ODN-**7**)

Figure S15. Thermal denaturation curves of duplexes a) ODN-**3**•ODN-**11**; b) ODN-**1**•ODN-**11**; c) ODN-**3**•ODN-7; d) ODN-**1**•ODN-**7** measured with 5 µM + 5 µM single-strand concentration in 100 mM NaCl, 10 mM $MgCl_2$, 10 mM Na-cacodylate buffer (pH = 7.0) at 260 nm.

pK_a Determination of nucleosides 1 and 2 by UV

Nucleoside **1** and **2** were dissolved in 0.1 M sodium phosphate (NaH_2PO_4) buffer, pH 7 (250 mL). Concentrated phosphorus acid was used to adjust the pH value of the buffer. At defined pH values, the UV absorbance of nucleosides was measured (Figure S16).

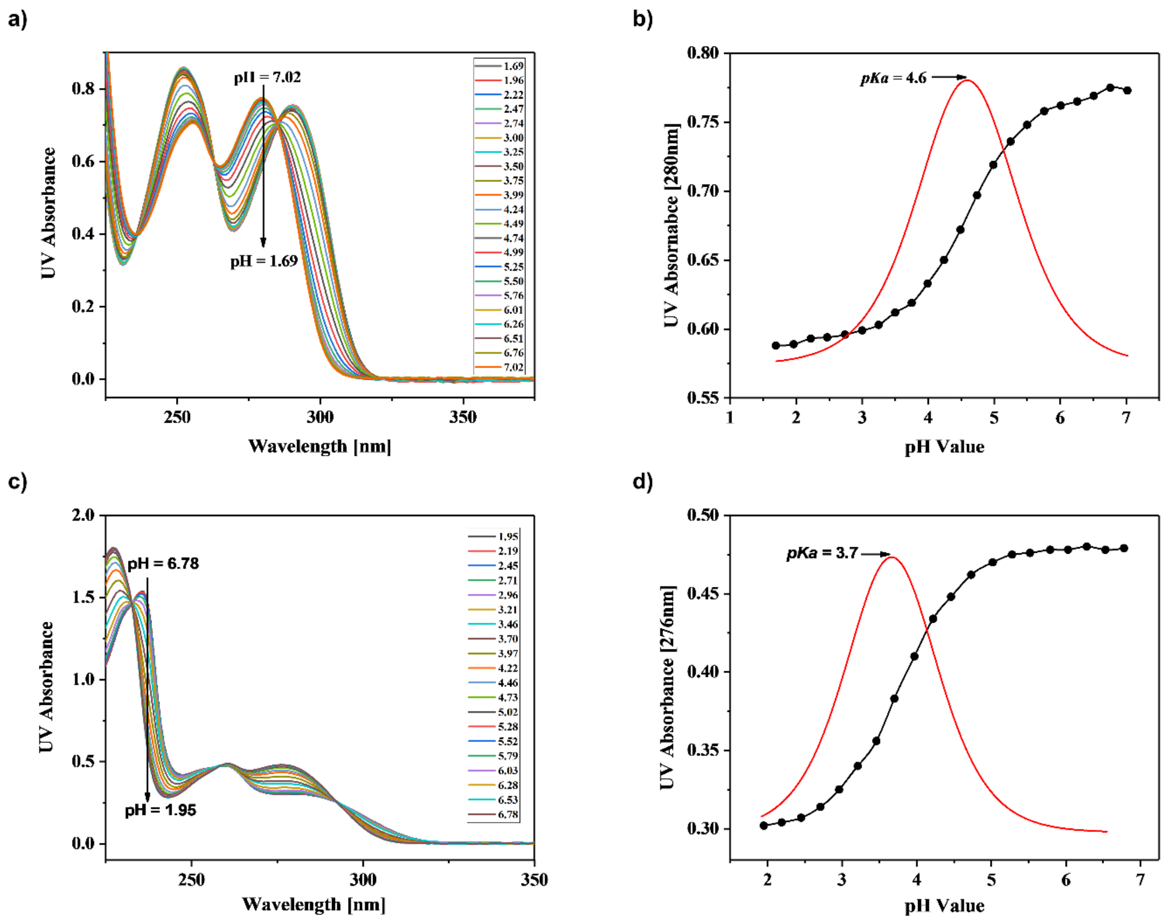

Figure S16. a) UV spectroscopic change of nucleoside **1** at various pH values; b) absorbance at 280 nm *vs* pH-value and its first derivative using data from (a). c) UV spectroscopic change of nucleoside **2** at various pH values; b) absorbance at 276 nm *vs* pH-value and its first derivative using data from (c). The cell path length of the cuvette was 1 cm.

UV-spectra of dA, 1 and 2

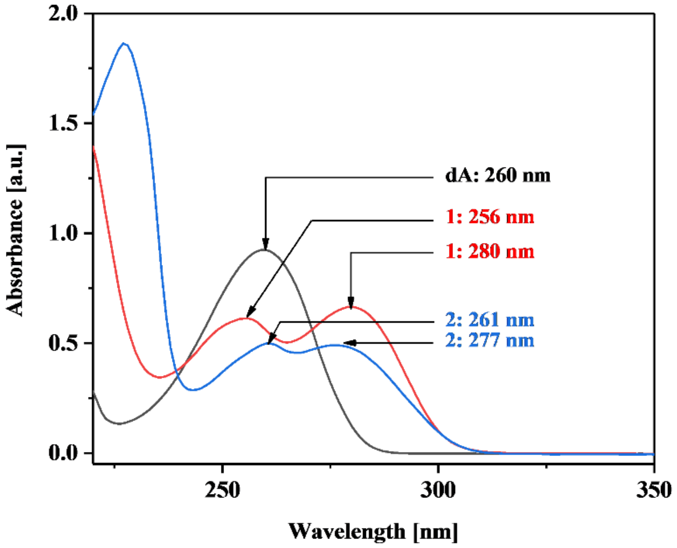

Figure S17. UV-spectra of dA (black), purine-2,6-diamine nucleoside **1** (red) and 8-aza-7-deaza-7-bromopurine-2,6-diamine nucleoside **2** (blue) measured with a concentration of 60 µM in H_2O. The cell path length of the cuvette was 1 cm.

CD-spectra of modified and unmodified single-stranded oligonucleotides

a)

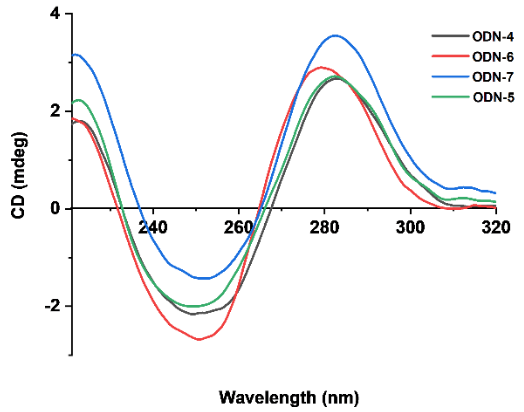

β-5’-d(AGT**1**TTGACCTA) (ODN-**4**)
β-5’-d(AGTATTG**1**CCTA) (ODN-**5**)
β-5’-d(AGT**1**TTG**1**CCTA) (ODN-**6**)
β-5’-d(**1**GT**1**TTG**1**CCTA) (ODN-**7**)

b)

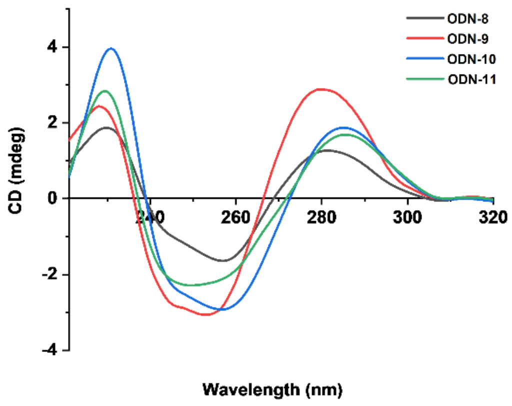

β-5’-d(AGT**2**TTGACCTA) (ODN-**8**)
β-5’-d(AGTATTG**2**CCTA) (ODN-**9**)
β-5’-d(AGT**2**TTG**2**CCTA) (ODN-**10**)
β-5’-d(**2**GT**2**TTG**1**CCTA) (ODN-**11**)

c)

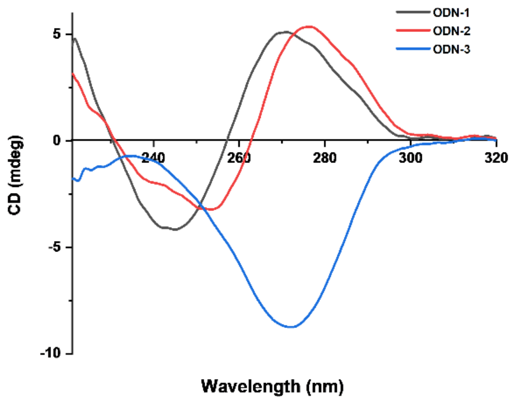

β-5’-d(TAGGTCAATACT) (ODN-**1**)
β-5’-d(AGTATTGACCTA) (ODN-**2**)
α-5’-d(TCATAACTGGAT) (ODN-**3**)

Figure S18. CD-spectra of a) ODN-**8**, ODN-**9**, ODN-**10** and ODN-**11** incorporating pyrazolo[3,4-*d*]pyrimidine-2,6-diamine nucleoside **2**; b) ODN-**4**, ODN-**5**, ODN-**6** and ODN-**7** incorporating purine-2,6-diamine nucleoside **1**; c) ODN-**1**, ODN-**2**, ODN-**3**. All measurements were performed in 100 mM NaCl, 10 mM $MgCl_2$, 10 mM Na-cacodylate, pH 7.0. The cell path length of the cuvette for the CD-spectra was 5 mm for a) and b) and 1.0 cm for c).

CD-spectra of modified and unmodified heterochiral and homochiral DNA duplexes

a)

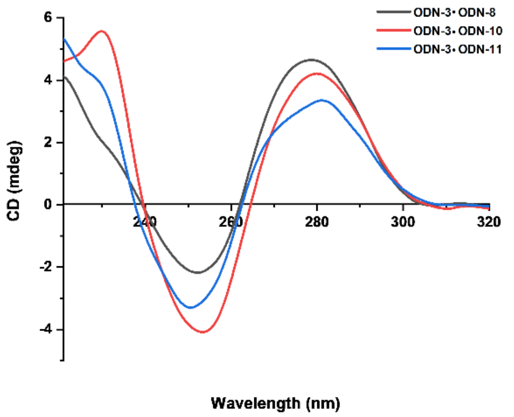

α-5’-d(TCATAACTGGAT) (ODN-**3**)
β-5’-d(AGT2TTGACCTA) (ODN-**8**)

α-5’-d(TCATAACTGGAT) (ODN-**3**)
β-5’-d(AGT2TTG2CCTA) (ODN-**10**)

α-5’-d(TCATAACTGGAT) (ODN-**3**)
β-5’-d(2GT2TTG2CCTA) (ODN-**11**)

b)

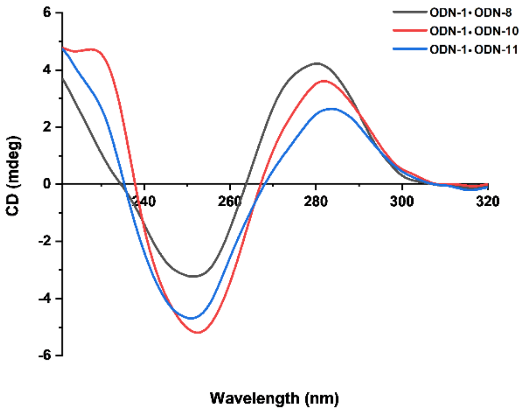

β-5’-d(TAGGTCAATACT) (ODN-**1**)
β-3’-d(ATCCAGTT2TGA) (ODN-**8**)

β-5’-d(TAGGTCAATACT) (ODN-**1**)
β-3’-d(ATCC2 GTT 2TGA) (ODN-**10**)

β-5’-d(TAGGTCAATACT) (ODN-**1**)
β-3’-d(ATCC2 GTT2 TG2) (ODN-**11**)

c)

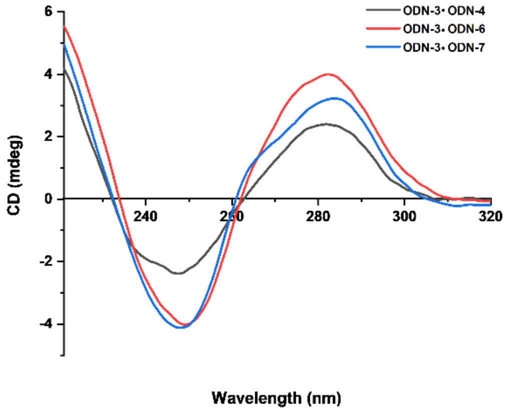

α-5’-d(TCATAACTGGAT) (ODN-**3**)
β-5’-d(AGT1TTGACCTA) (ODN-**4**)

α-5’-d(TCATAACTGGAT) (ODN-**3**)
β-5’-d(AGT1TTG 1 CCTA) (ODN-**6**)

α-5’-d(TCATAACTGGAT) (ODN-**3**)
β-5’-d(1GT 1TTG 1CCTA) (ODN-7)

d)

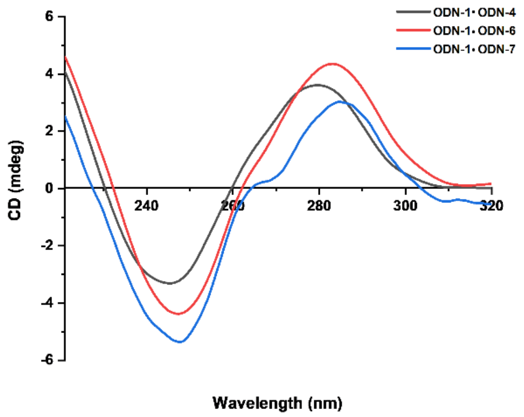

β-5’-d(TAGGTCAATACT) (ODN-**1**)
β-3’-d(ATCCAGTT1TGA) (ODN-**4**)

β-5’-d(TAGGTCAATACT) (ODN-**1**)
β-3’-d(ATCC 1GTT 1TGA) (ODN-**6**)

β-5’-d(TAGGTCAATACT) (ODN-**1**)
β-3’-d(ATCC1 GTT 1TG1) (ODN-7)

Figure S19. CD-spectra of a) the heterochiral duplexes ODN-**3**•ODN-**8**, ODN-**3**•ODN-**10**, ODN-**3**•ODN-**11** incorportaing **2**; b) the homochiral duplexes. b) ODN-**1**•ODN-**8**, ODN-**1**•ODN-**10**, ODN-**1**•ODN-**11** incorporating **2**; c) the heterochiral duplexes ODN-**3**•ODN-**4**, ODN-**3**•ODN-**6**, ODN-**3**•ODN-**7** incorporating **1**; d) the homochiral duplexes ODN-**1**•ODN-**4**, ODN-**1**•ODN-**6**, ODN-**1**•ODN-**7** incorporating **1**. All measurements were performed in 100 mM NaCl, 10 mM $MgCl_2$, 10 mM Na-cacodylate, pH 7.0. The cell path length of the cuvette for the CD-spectra was 5 mm.

CD-spectra of modified and unmodified single-stranded oligonucleotides and heterochiral and homochiral DNA duplexes (calculated and measured) incorporating the purine-2,6-diamine nucleoside 1

a)

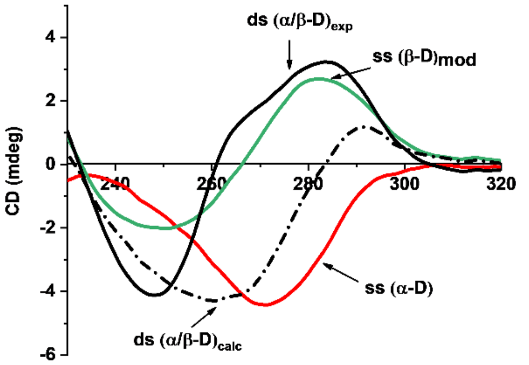

α-5'-d(TCATAACTGGAT) (ODN-**3**, ss (α-D), red)
β-5'-d(**1**GT**1**TTG**1**CCTA) (ODN-**7**, ss (β-D$_{mod}$) green)
ds (α/β-D)$_{exp}$: ODN-**3**•ODN-**7** measured (black)
ds (α/β-D)$_{calc}$: ODN-**3**•ODN-**7** calculated (black, dashed)

b)

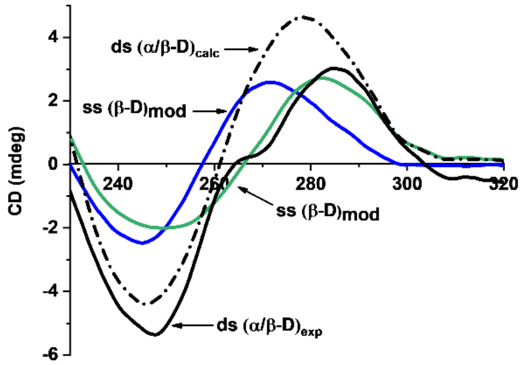

β-5'-d(TAGGTCAATACT) (ODN-**1**, ss (β-D), blue)
β-3'-d(ATCC**1** GTT**1** TG**1**) (ODN-**7**, ss (β-D), green)
ds (β/β-D)$_{exp}$: ODN-**1**•ODN-**7** measured (black)
ds (β/β-D)$_{calc}$: ODN-**1**•ODN-**7** calculated (black, dashed)

Figure S20. CD-spectra of a) α-D oligonucleotide ODN-**3**, β-D oligonucleotide ODN-**7** (3 incorporations of **1**), duplex ODN-**3**•ODN-**7** and the calculated CD-spectra (from the sum of the CD-spectra of ODN-**3** and ODN-**7**) of duplex ODN-**3**•ODN-**7**. b) CD-spectra of a) β-D oligonucleotide ODN-**1**, β-D oligonucleotide ODN-**7** (3 incorporations of **1**), duplex ODN-**1**•ODN-**7** and the calculated CD-spectra (from the sum of the CD-spectra of ODN-**1** and ODN-**7**) of duplex ODN-**3**•ODN-**7**. All measurements were performed in 100 mM NaCl, 10 mM $MgCl_2$, 10 mM Na-cacodylate, pH 7.0. The cell path length of the cuvette for the CD-spectra was 5 mm.

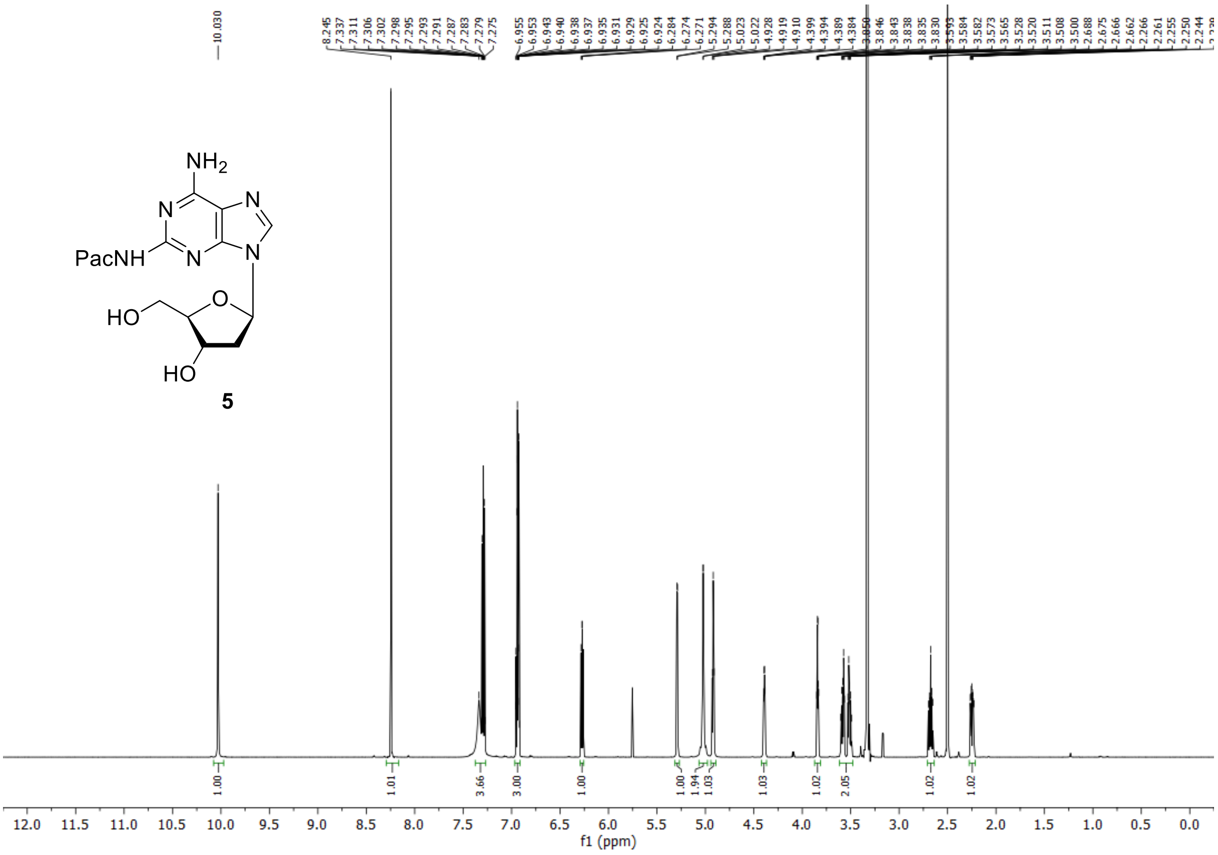

Figure S21. ^{1}H NMR spectrum of compound 5

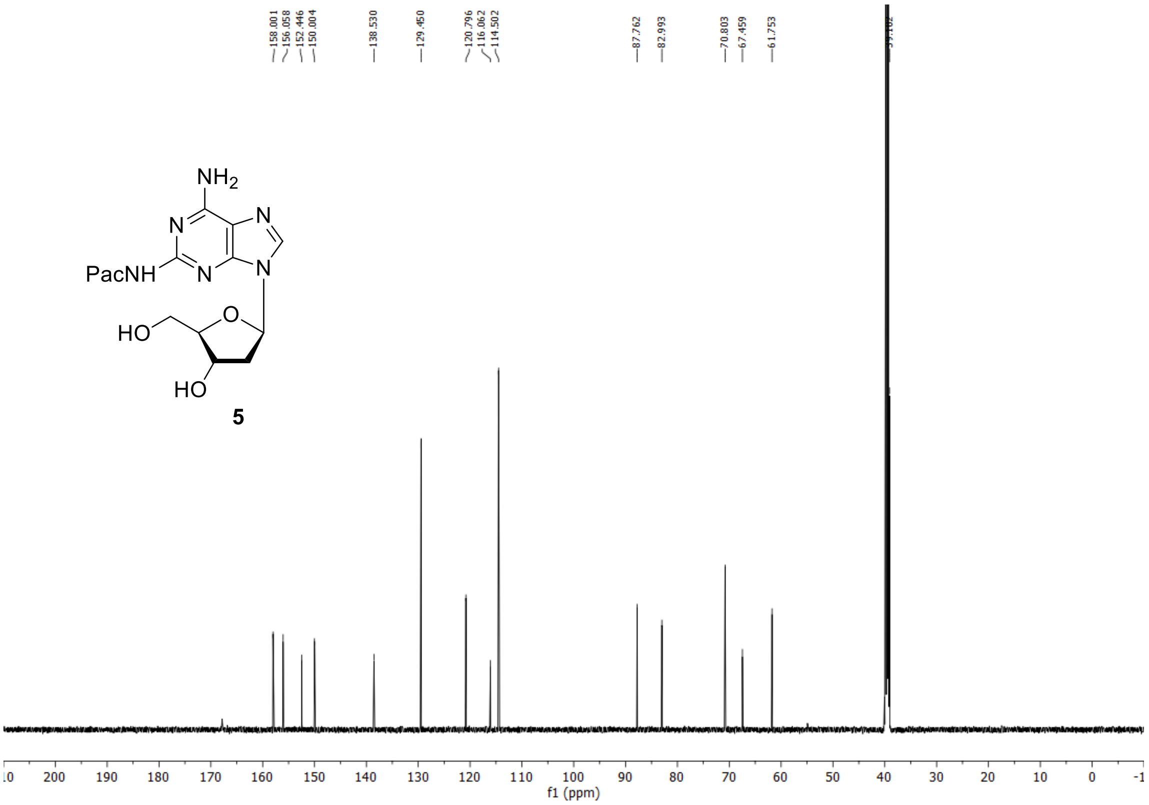

Figure S22. ^{13}C NMR spectrum of compound 5

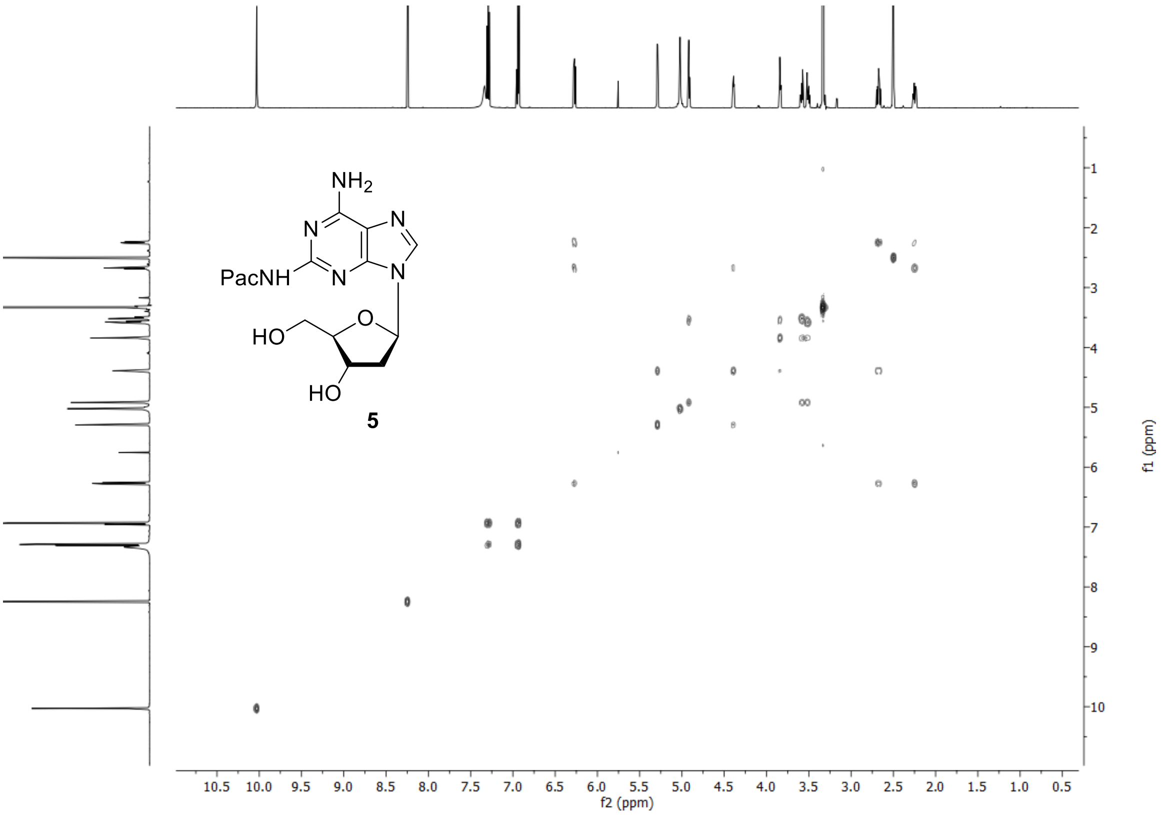

Figure S23. COSY spectrum of compound 5

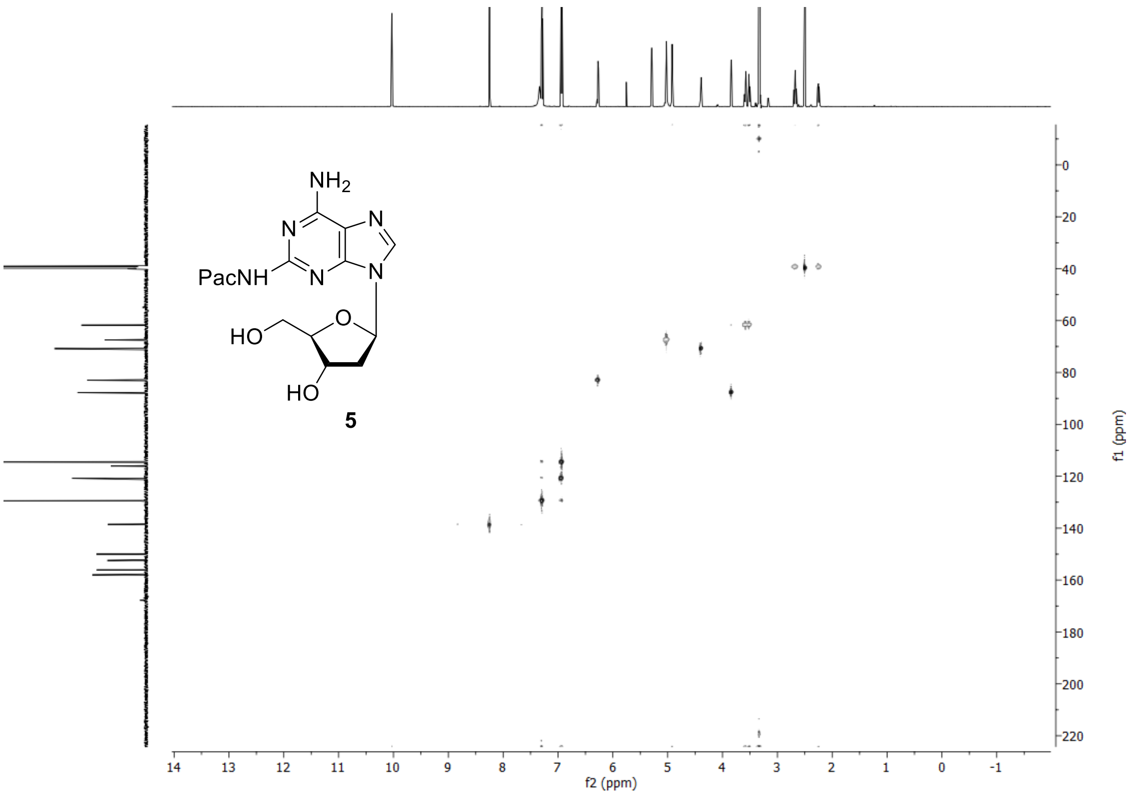

Figure S24. HSQC spectrum of compound 5

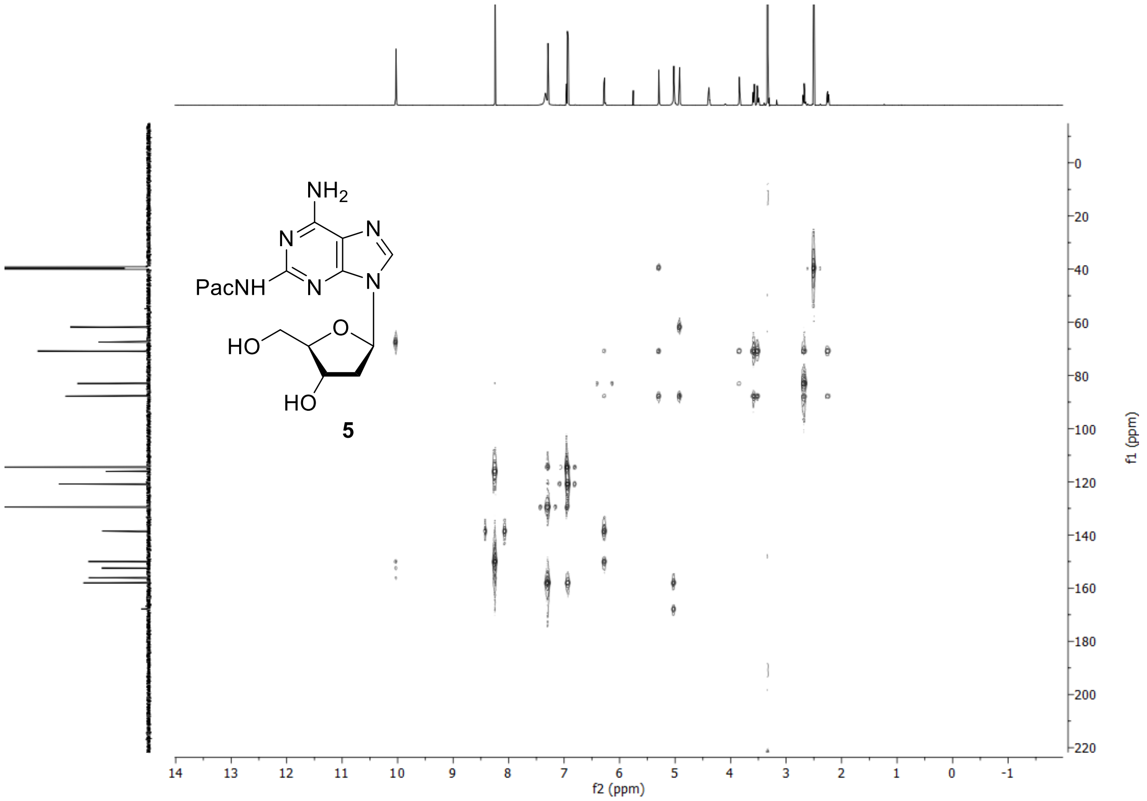

Figure S25. HMBC spectrum of compound 5

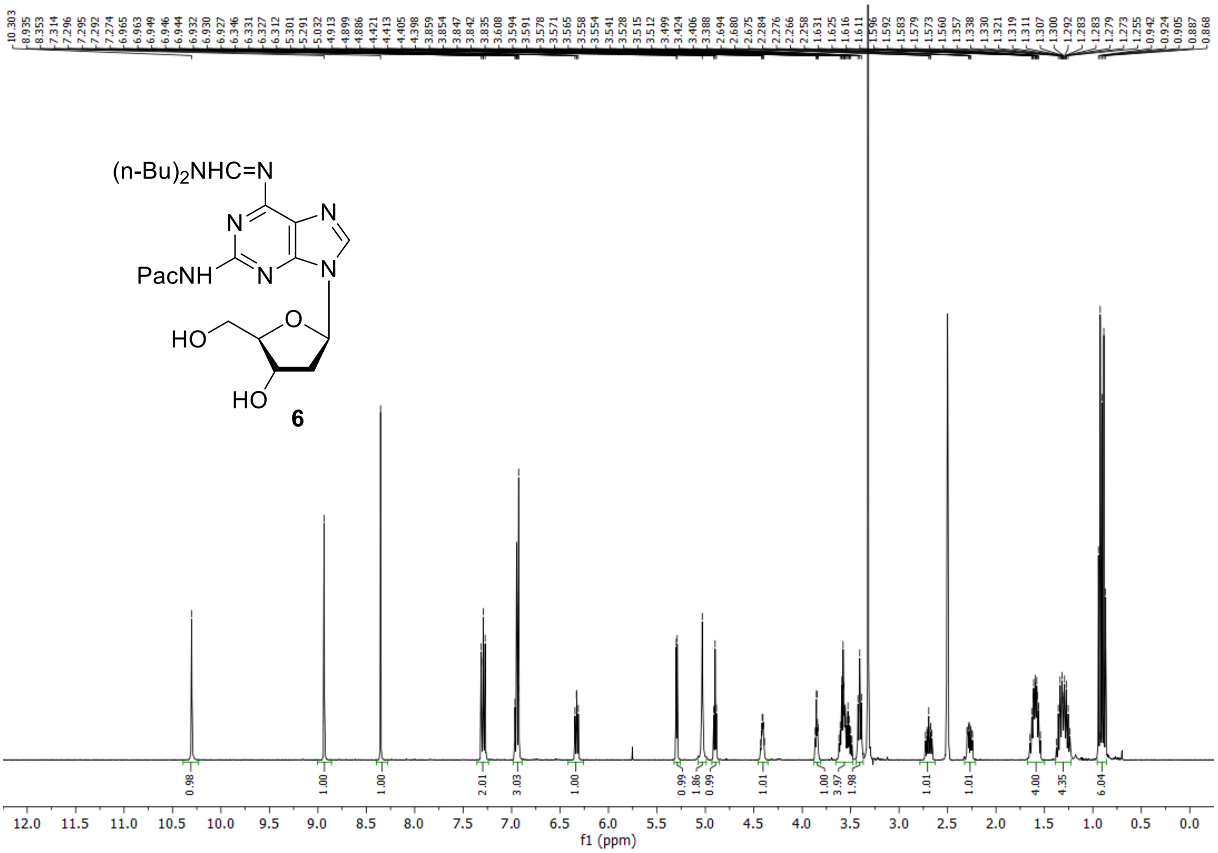

Figure S26. ^{1}H NMR spectrum of compound 6

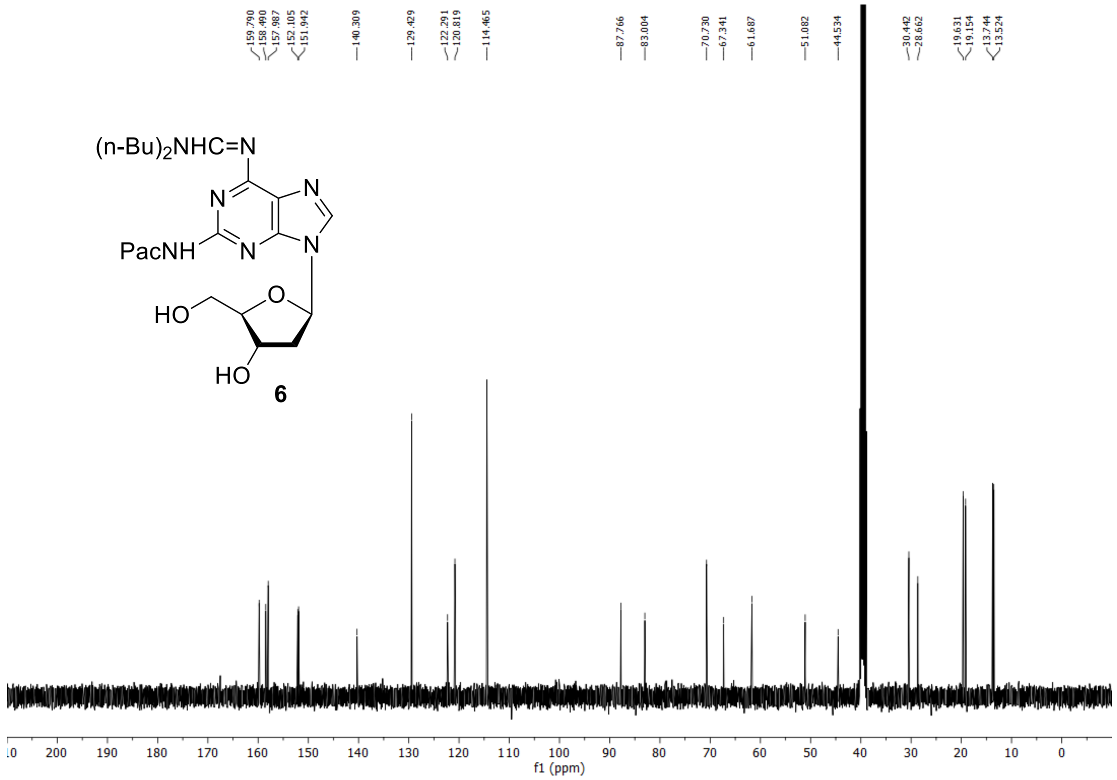

Figure S27. ^{13}**C NMR spectrum of compound 6**

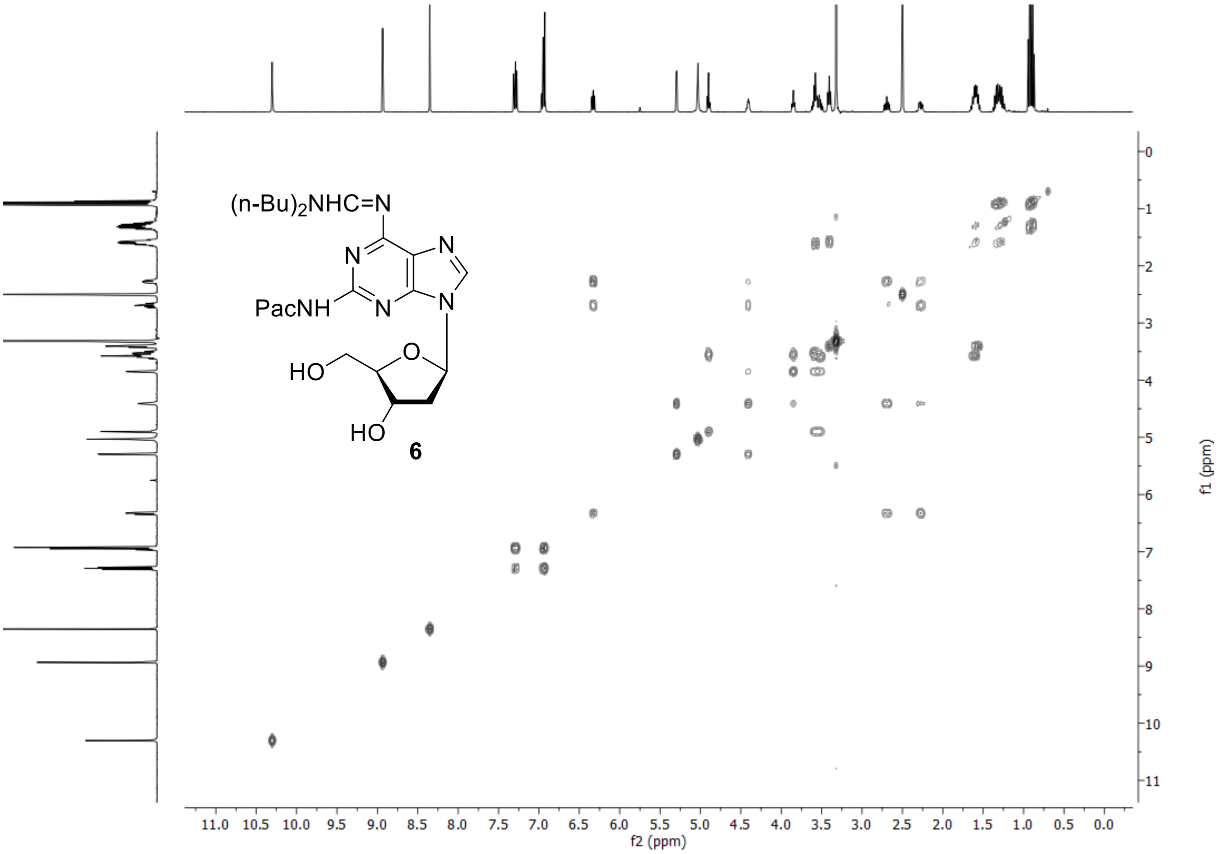

Figure S28. COSY spectrum of compound 6

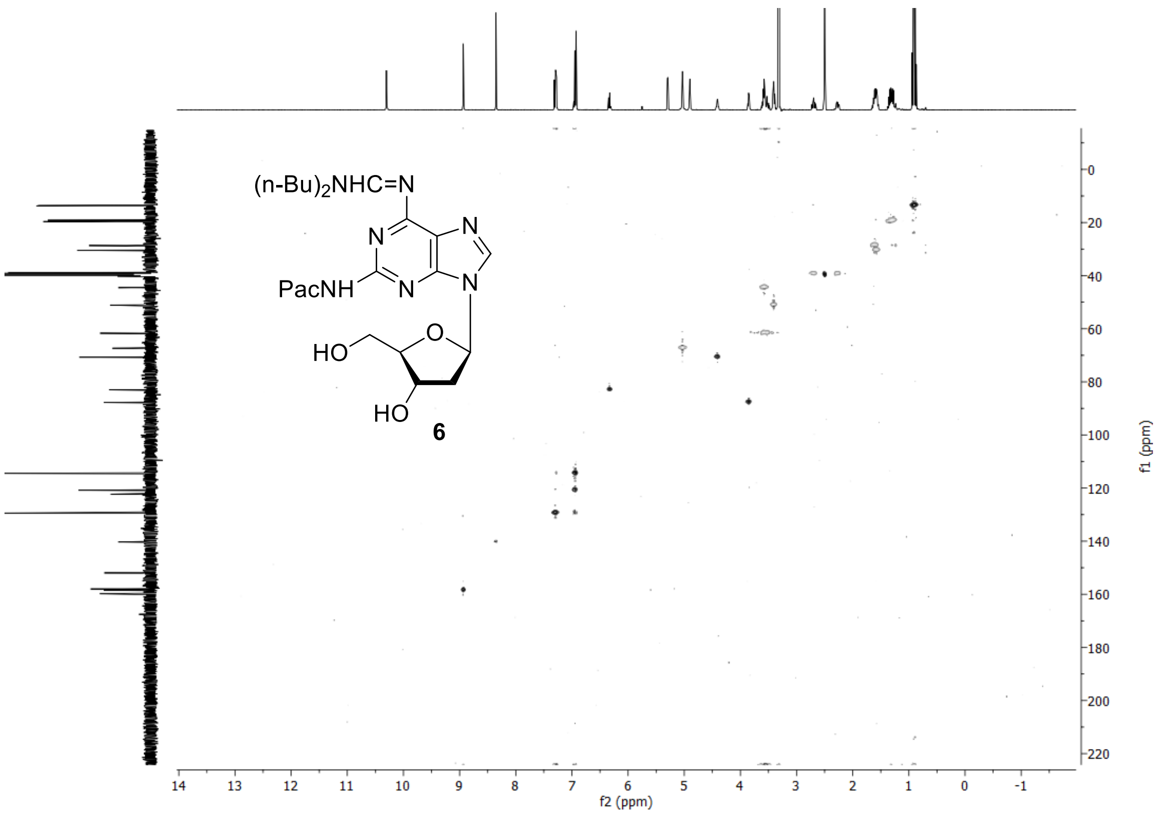

Figure S29. HSQC spectrum of compound 6

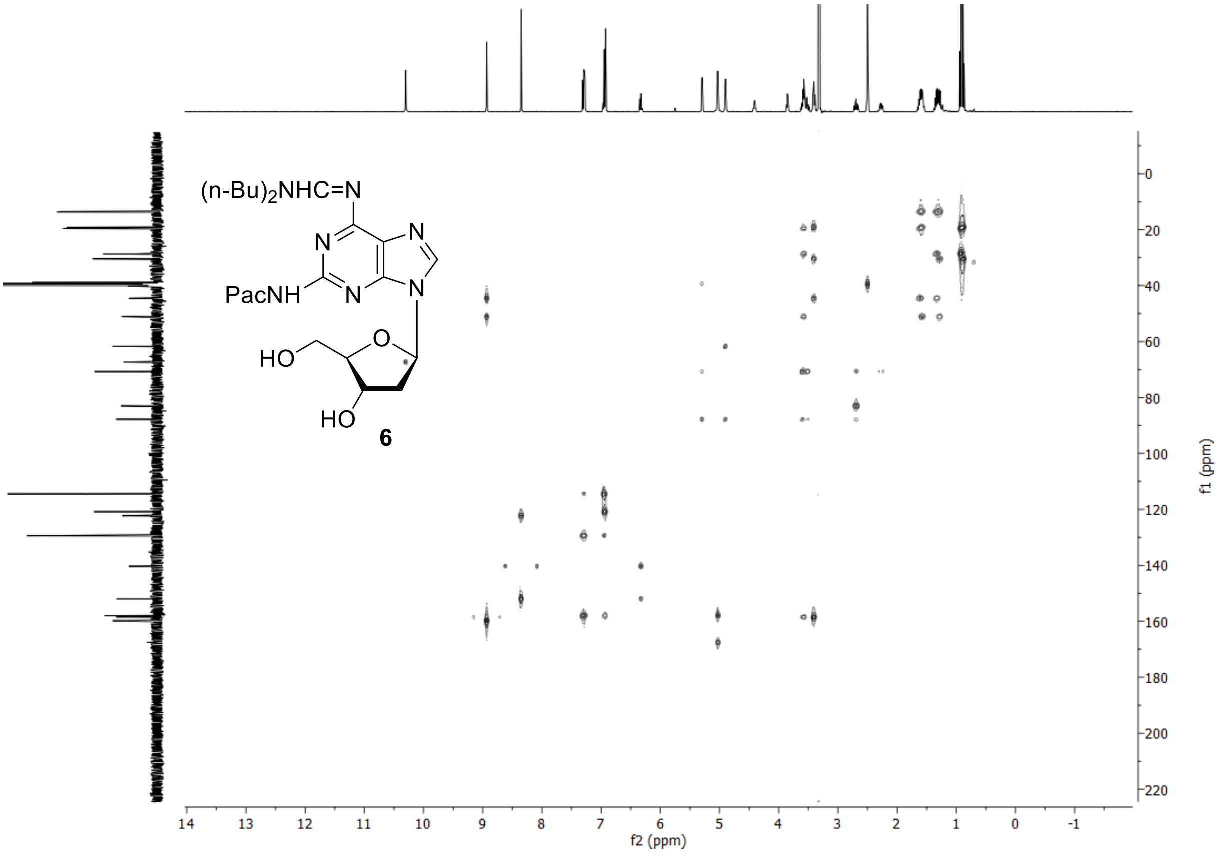

Figure S30. HMBC spectrum of compound 6

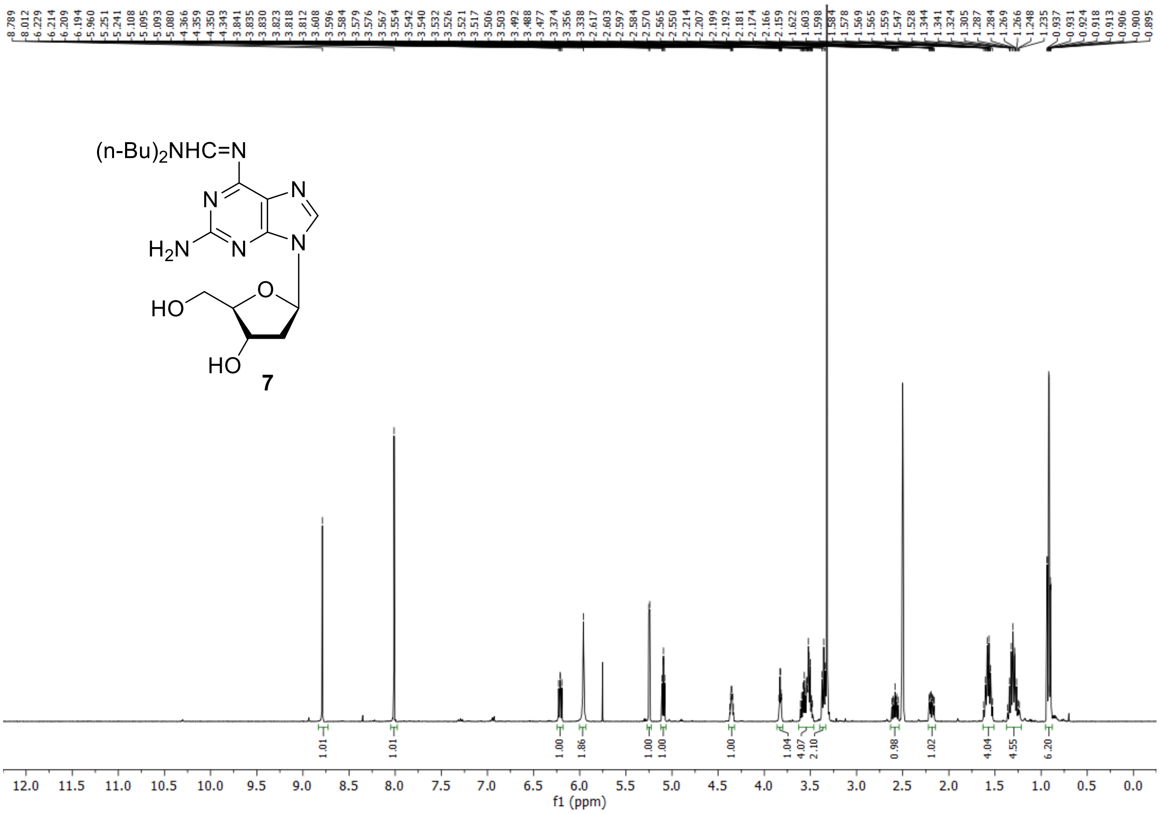

Figure S31. ^{1}H NMR spectrum of compound 7

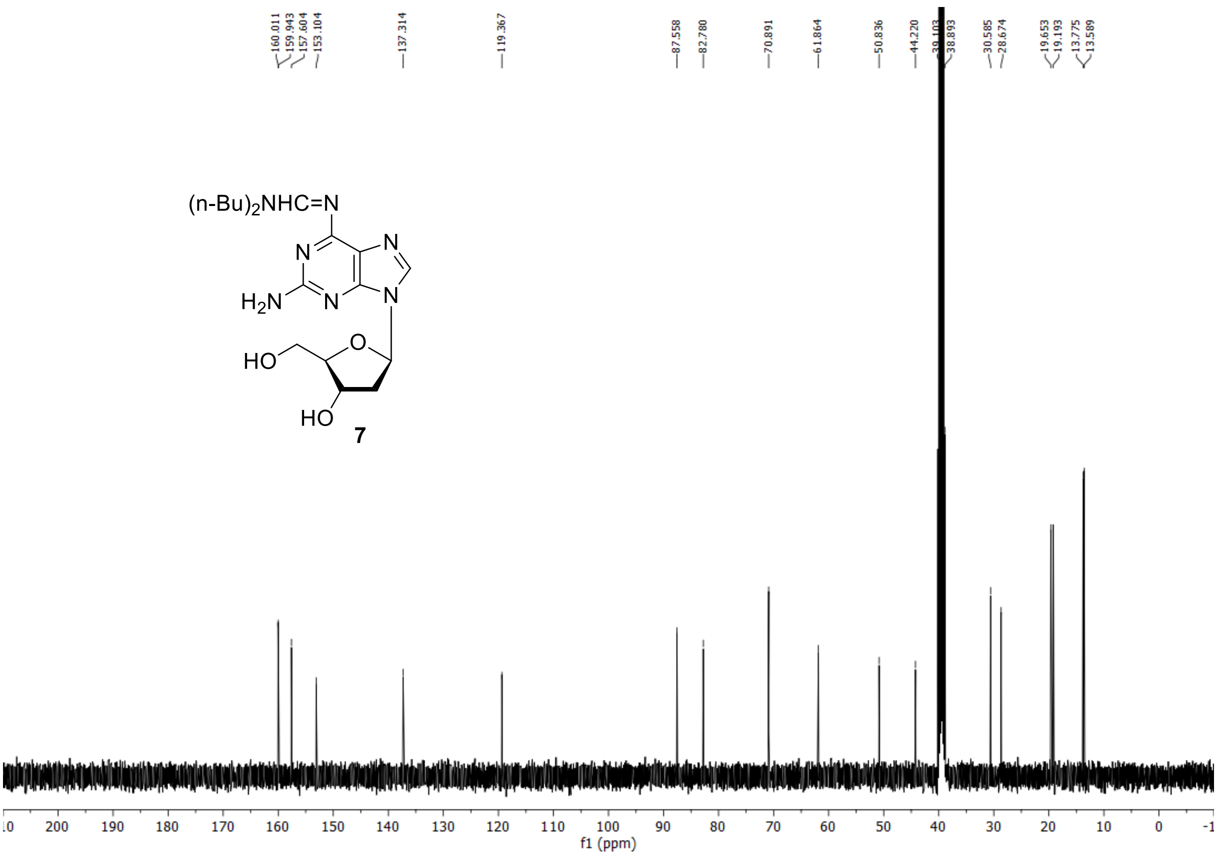

Figure S32. ^{13}C NMR spectrum of compound 7

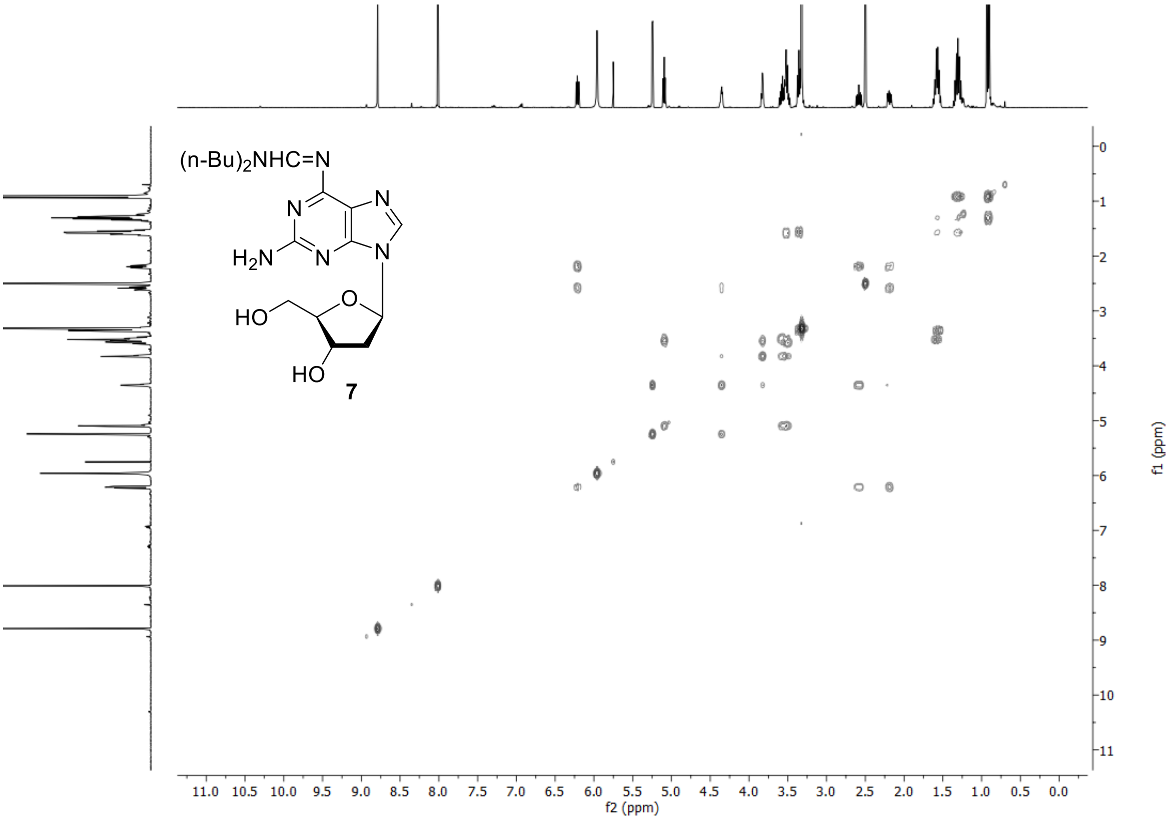

Figure S33. COSY spectrum of compound 7

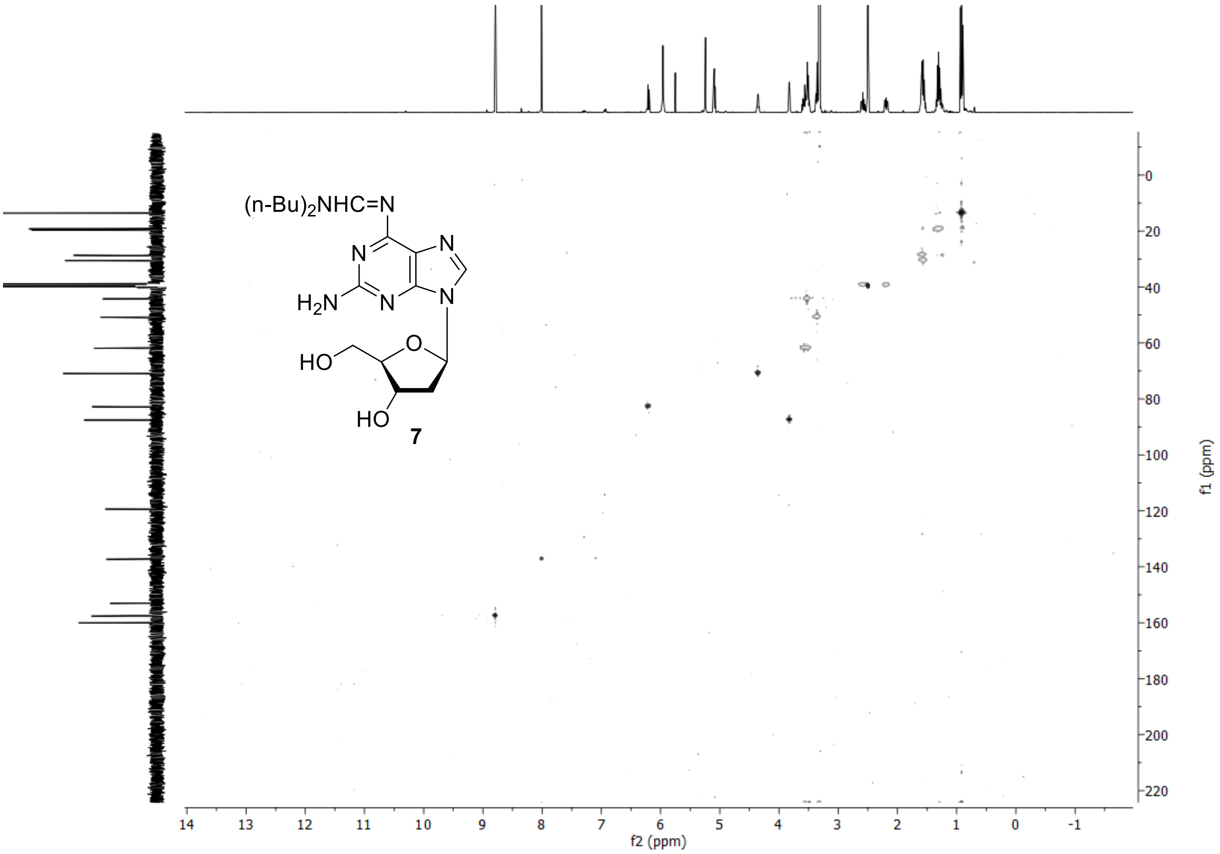

Figure S34. HSQC spectrum of compound 7

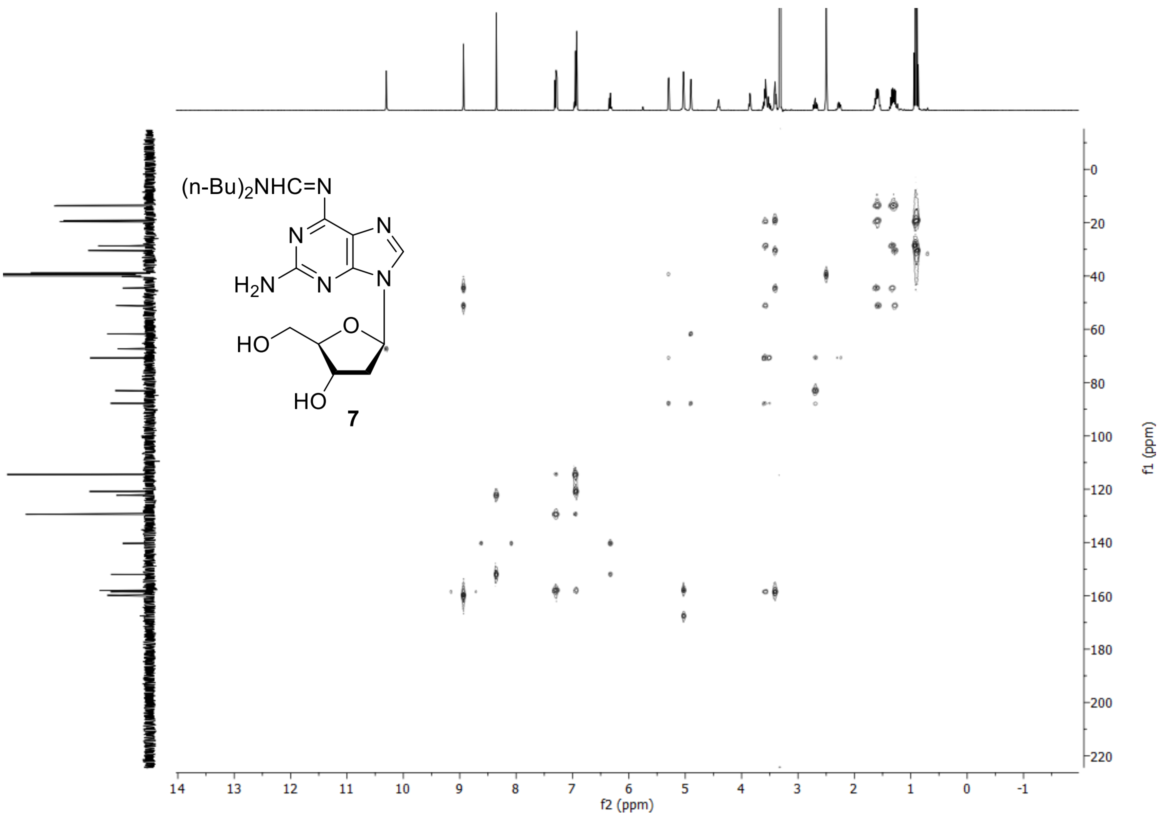

Figure S35. HMBC spectrum of compound 7

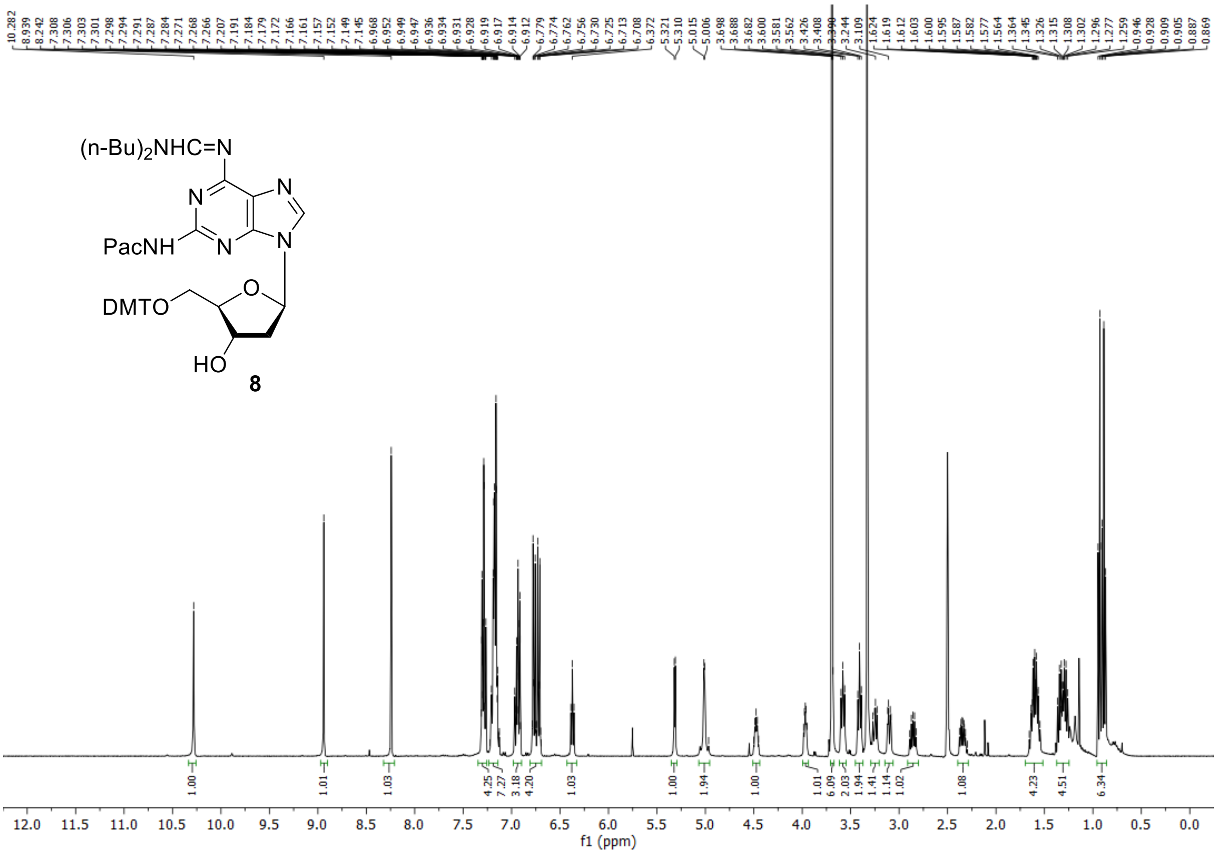

Figure S36. ^{1}H NMR spectrum of compound 8

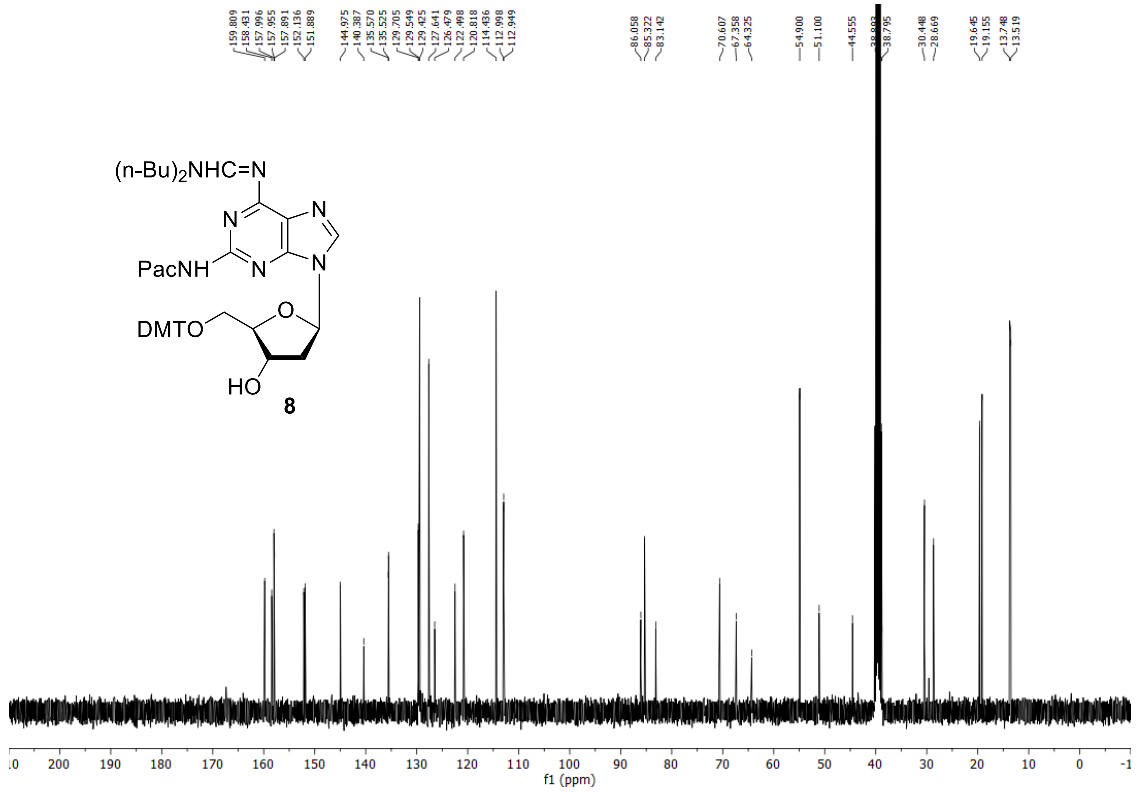

Figure S37. ^{13}C **NMR spectrum of compound 8**

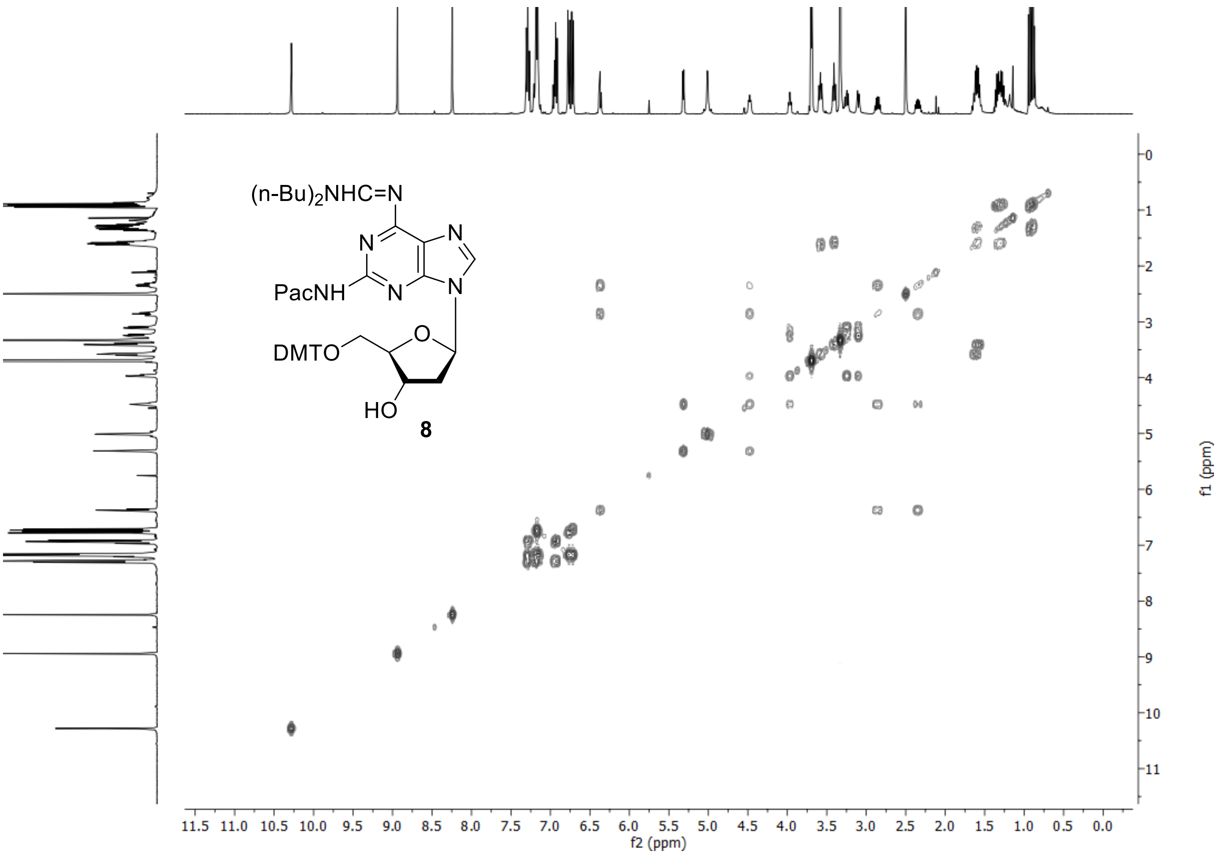

Figure S38. COSY spectrum of compound 8

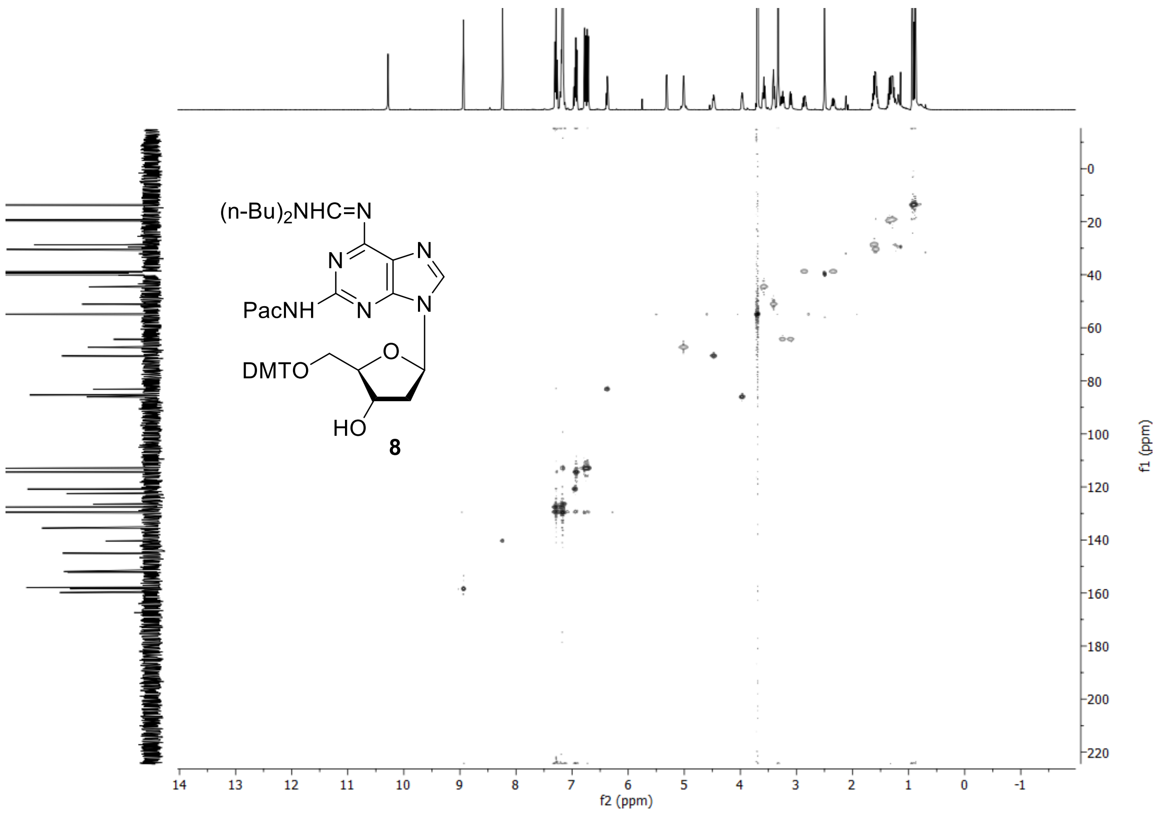

Figure S39. HSQC spectrum of compound 8

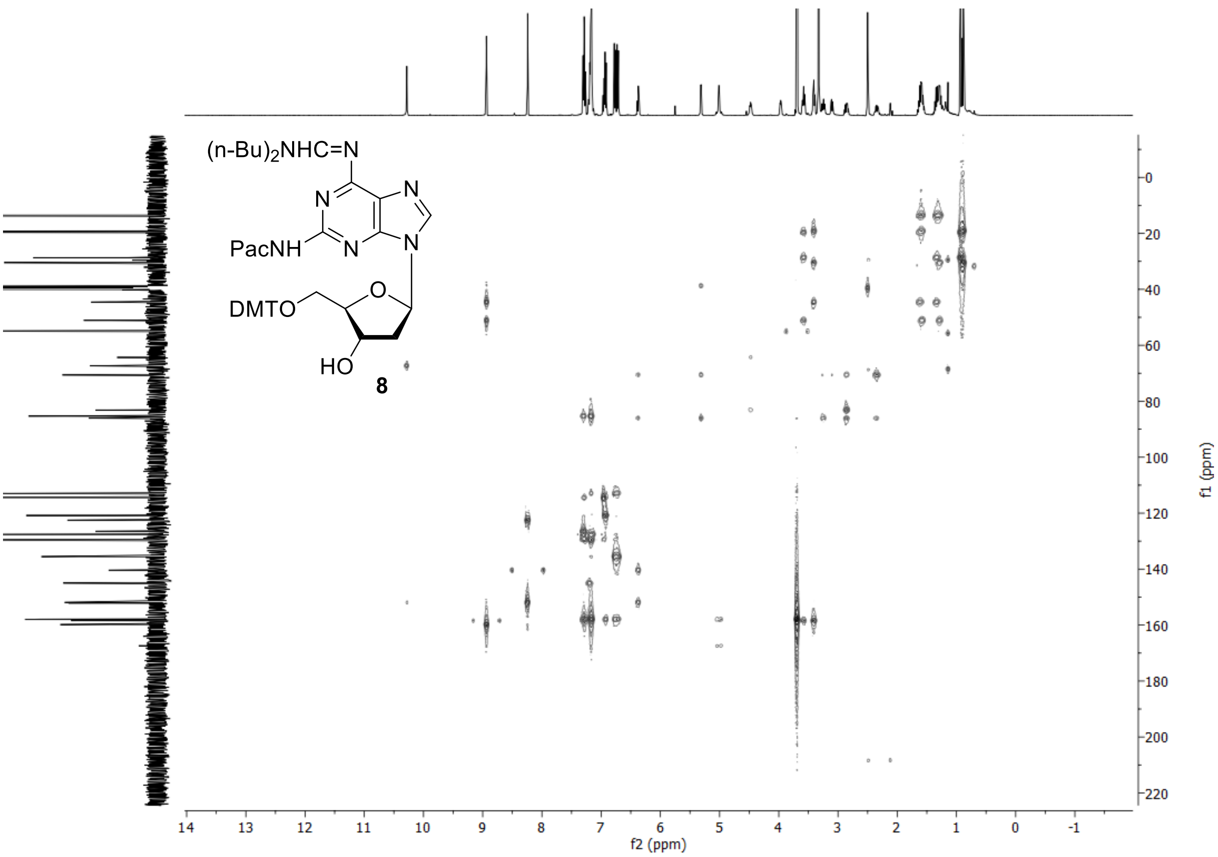

Figure S40. HMBC spectrum of compound 8

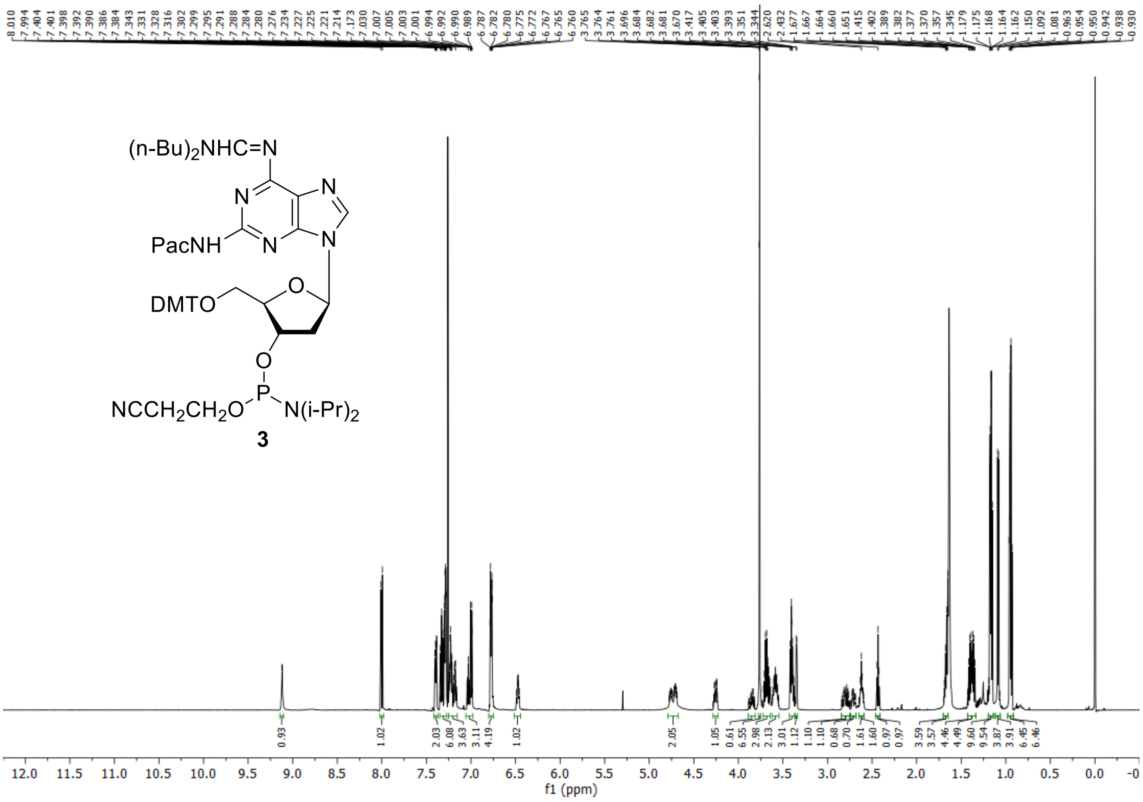

Figure S41. ^{1}H NMR spectrum of compound 3

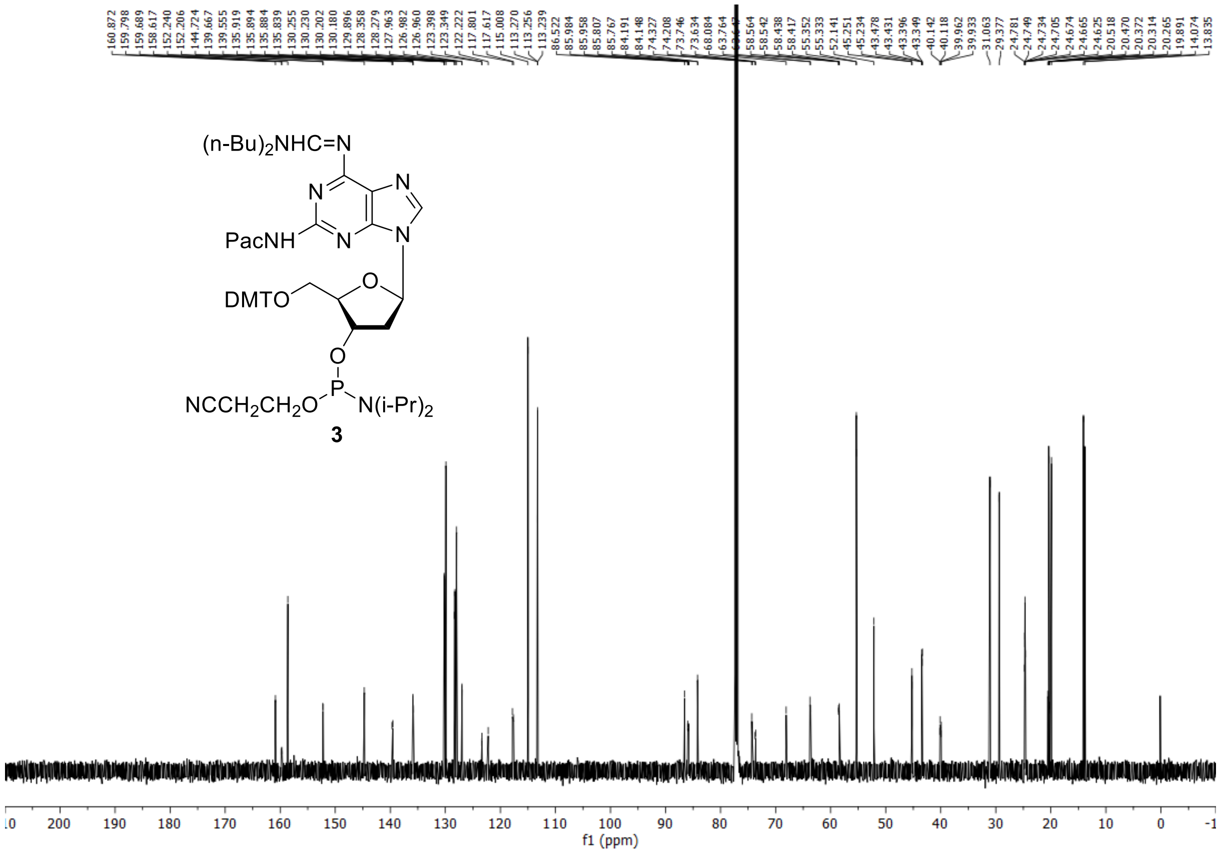

Figure S42. ^{13}C NMR spectrum of compound 3

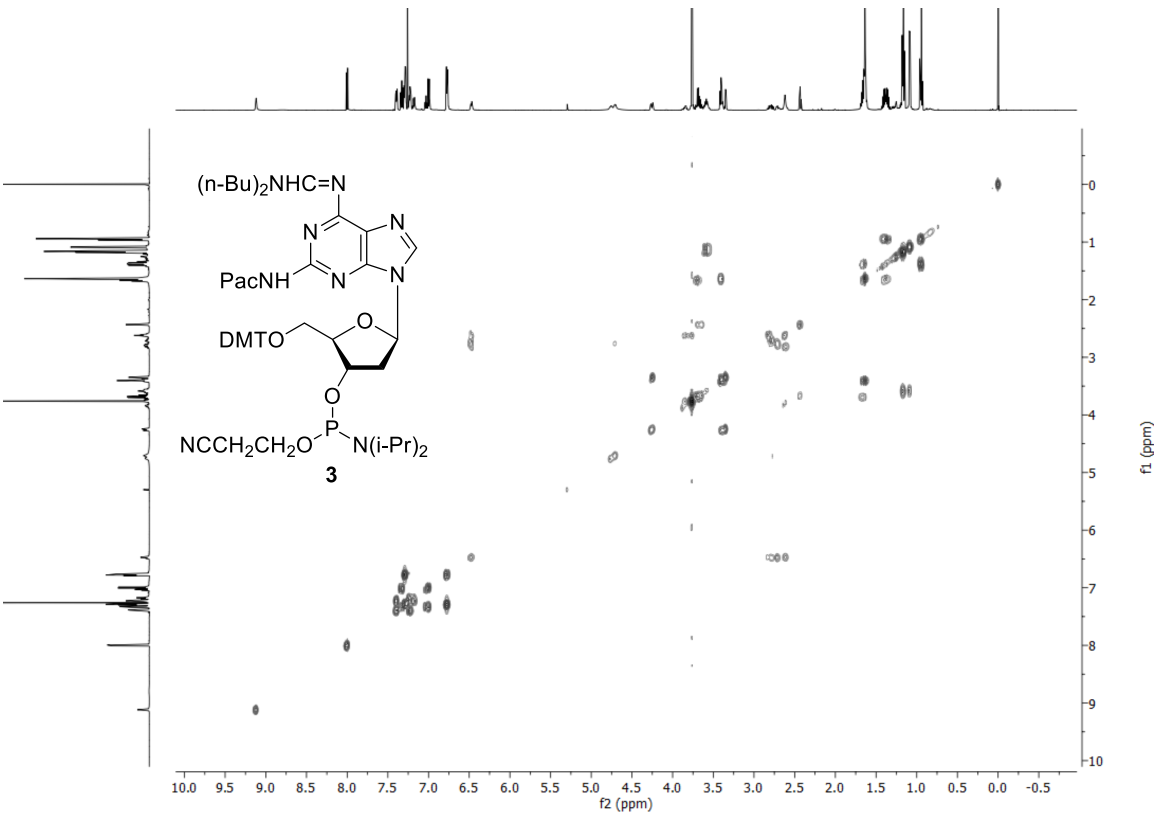

Figure S43. COSY spectrum of compound 3

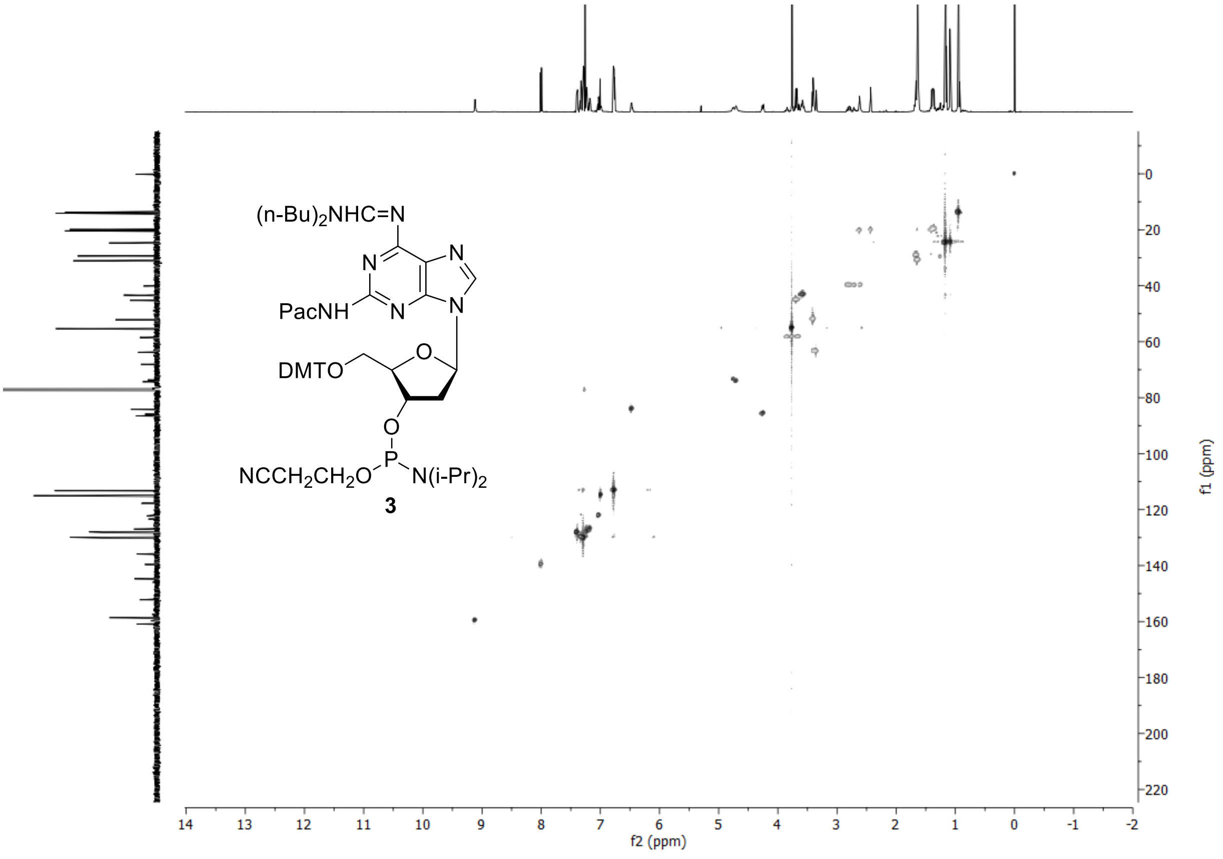

Figure S44. HSQC spectrum of compound 3

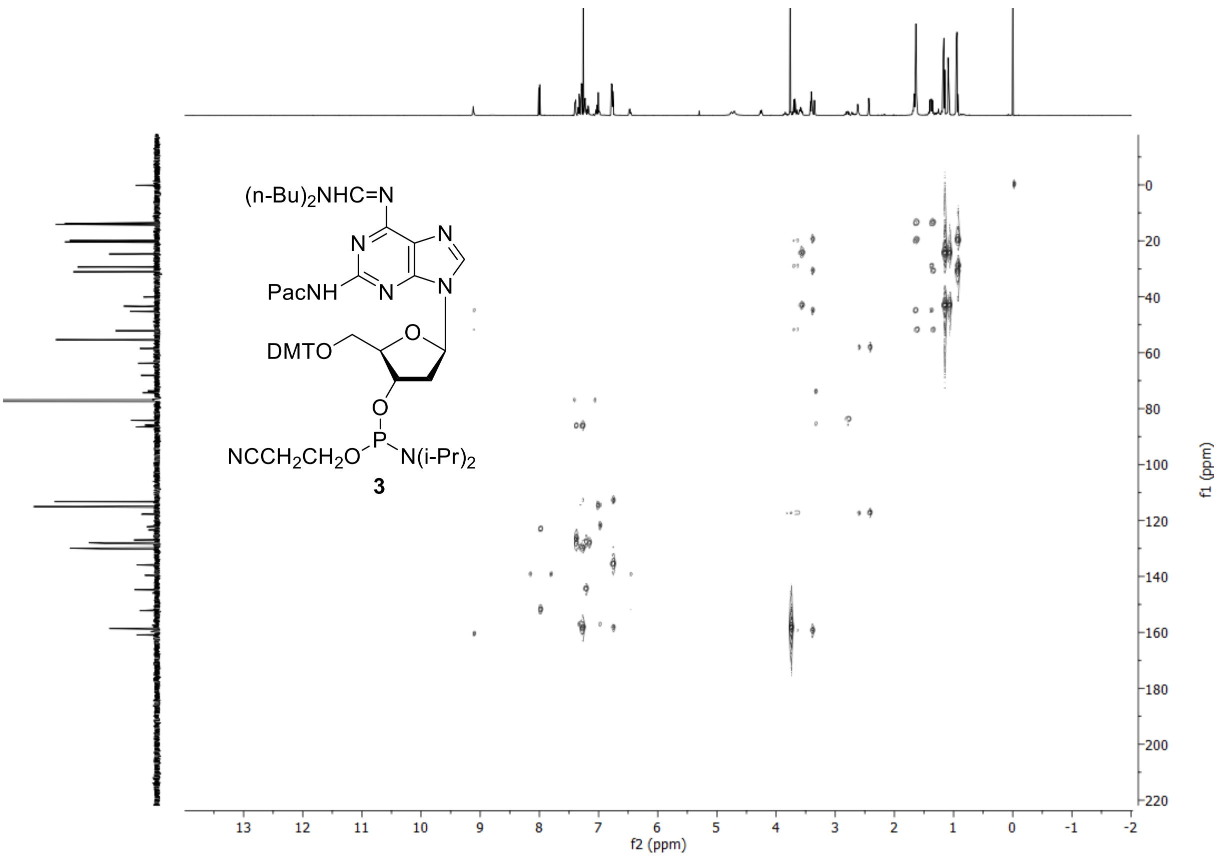

Figure S45. HMBC spectrum of compound 3

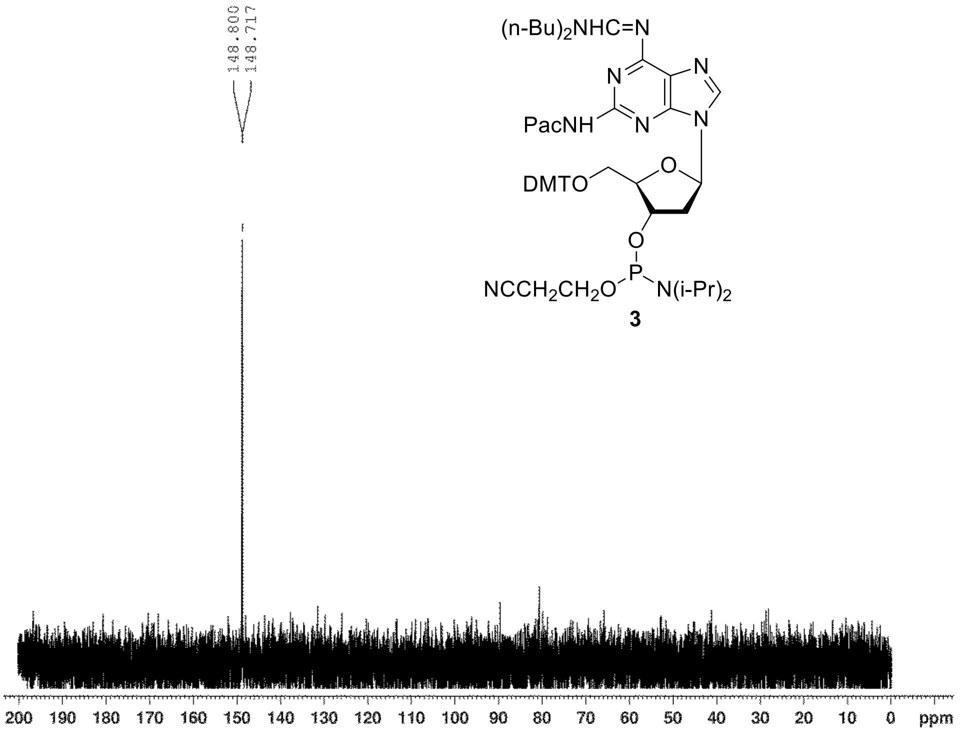

Figure S46. ^{31}P-NMR spectrum of compound 3
